# Combination of Chemically-Characterized Essential Oils from *Eucalyptus polybractea*, *Ormenis mixta,* and *Lavandula burnatii*: Optimization of a New Complete Antibacterial Formulation Using Simplex-Centroid Mixture Design

**DOI:** 10.1155/2023/5593350

**Published:** 2023-08-21

**Authors:** Mohamed Jeddi, Naoufal El Hachlafi, Mouhcine Fadil, Nesrine Benkhaira, Samir Jeddi, Zineb Benziane Ouaritini, Kawtar Fikri-Benbrahim

**Affiliations:** ^1^Laboratory of Microbial Biotechnology and Bioactive Molecules, Sciences and Technologies Faculty, Sidi Mohamed Ben Abdellah University, P.O. Box 2202, Imouzzer Road, Fez, Morocco; ^2^Laboratory of Natural Substances, Pharmacology, Environment, Modeling, Health and Quality of Life (SNAMOPEQ), Faculty of Sciences, Sidi Mohamed Ben Abdellah University, Fez 30 000, Morocco; ^3^Laboratory of Applied Organic Chemistry, Sidi Mohamed Ben Abdellah University, P.O. Box 2202, Road of Imouzzer, Fez, Morocco

## Abstract

This study aims to identify the volatile profile of three essential oils obtained from *Eucalyptus polybractea cryptonifera* (EPEO), *Ormenis mixta* (OMEO), and *Lavandula burnatii briquet* (LBEO) and to examine their combined antibacterial activity that affords the optimal inhibitory ability against *S. aureus* and *E. coli* using simplex-centroid mixture design and checkerboard assay. Essential oils (EOs) were isolated by hydrodistillation and characterized using gas chromatography-mass spectrometry (GC-MS) and gas chromatography coupled with flame-ionization detector (GC-FID). The antibacterial activity was performed using disc diffusion and microdilution assays. The chemical analysis revealed that 1,8-cineole (23.75%), p-cymene (22.47%), and *α*-pinene (11.20%) and p-menthane-1,8-diol (18.19%), *α*-pinene (10.81%), and D-germacrene (9.17%) were the main components detected in *E. polybractea* and *O. mixta* EOs, respectively. However, *L. burnatii* EO was mainly represented by linalool (24.40%) and linalyl acetate (18.68%). The EPEO, LBEO, and OMEO had a strong antibacterial effect on *S. aureus* with minimal inhibitory concentrations (MICs) values ranging from 0.25 to 0.5% (v/v). Furthermore, the combination of 1/2048 MIC_EPEO_ + 1/4 MIC_LBEO_ showed a synergistic antibacterial effect on *S. aureus* with a FIC index of 0.25, while the formulation of 1/4 MIC_EPEO_ + 1/4 MIC_OMEO_ demonstrated an antibacterial synergistic activity on *E. coli* with a FIC index of 0.5. Moreover, the simplex-centroid mixture design reported that the most effective combinations on *E. coli* and *S. aureus* correspond to 32%/28%/40% and 35%/30%/35% of *E. polybractea*, *O. mixta,* and *L. burnatii,* respectively. Presented information highlights the action of antibacterial formulations of these EOs and suggests their potential applications as alternatives to commercialized drugs to contract the development of bacteria causing serious infections and food deterioration.

## 1. Introduction

Infectious diseases triggered by antimicrobial resistance (AMR) are among the key issues impacting morbidity and fatality in patients suffering from such problems. The AMR has become a main public health concern that threatens the effective treatment of a broad range of infections caused by bacteria, fungi, viruses, and parasites no longer vulnerable to the common antibiotics used to prevent them. For multiple decades, bacteria causing common or severe infections have developed resistance to each novel antibiotic coming to market. The impact of antimicrobial resistance concerning the public health charge is quite difficult to predict. The Center for Disease Control and Prevention (CDC) estimated that more than two million people per year are affected with antibiotic-resistant infections, with at least 23,000 deaths as a consequence of infectious diseases. In light of this fact, it is crucial to take action to prevent a developing healthcare crisis [1]. In fact, natural products represent a source of safe and effective agents which can be used as alternative to antimicrobial medications since their continuous use can increase the resistance of microorganisms, thus decreasing the efficiency of these drugs [2]. Essential oils (EOs) have been broadly employed for treating various ailments due to their famous antimicrobial activities [3]. Nowadays, EOs have been suggested and proved as antimicrobial products in complementary medicine. These properties are associated with the complex bioactive compounds of EOs, especially terpenes, aldehydes, phenylpropanoids, alcohols, esters, and ketones, which possess varied antimicrobial actions [4]. These bioactive molecules may act through different modes of action to apply their antimicrobial effect. Generally, EOs can induce membrane disruption and metabolic damages leading to cell death [5]. The application of EOs has been restricted due to their effects on sensory characteristics, specifically at elevated doses. Therefore, it is imperative to detect minimum inhibitory concentration for EOs and to evolve EO formulations in order to obtain synergistic effects, thus diminishing the amounts of EOs impacting the organoleptic properties without altering their antimicrobial effects [6]. Numerous works have established synergism between EOs using the checkerboard method without highlighting optimal EO formulations. However, only a few studies have recently used mixture design [7–10]. The mixture design approach can diminish the number of tests and predicted results can be modeled graphically and statistically aiming to minimize the total error. Through this approach, the accurate amounts of different EOs can be optimally associated to attain better mixtures [7]. In this context, we selected three medicinal plants to test the single and combined antibacterial effect of their EOs on pathogenic strains. These plants were chosen based on our previous ethnobotanical surveys which have demonstrated their therapeutic and culinary virtues [11–14]. In addition, some *in vitro* studies have revealed their significant antimicrobial abilities [8, 15, 16].

*Eucalyptus polybractea,* also named blue-leaved or blue mallee, is a multistemmed mallee eucalypt. This species belongs to the Myrtaceae family [17]. *Eucalyptus* has been used in traditional medicine to heal a variety of disorders including flu, fever, colds, sores, muscular pains, internal aches, and inflammation [18]. This plant oil is chiefly characterized by 1,8-cineole. The volatile oils derived from this genus are widely applied for pharmaceuticals, cosmetics, and food crops. Indeed, *Eucalyptus* oils have been reported to exhibit significant antibacterial, antifungal, anti-inflammatory, and antioxidant properties [15, 19].

*Ormenis mixta*, known as wild chamomile, is an endemic species of western and central Morocco. This plant belongs to the Asteraceae family and is usually used as general tonic, neurotonic, and aphrodisiac. It is endowed with anti-infectious, parasiticidal, antimutagenic, anticholesterol, and wound-healing properties [12]. It was previously reported that the plant oil is mainly characterized by D-germacrene and 1,8-cineole and possess promising antioxidant, anti-inflammatory, antidiabetic, and antimicrobial properties [20].

*Lavandula burnatii*, commonly known as Burnat's lavender, is a perennial herb native to the Mediterranean region and attributed to the Lamiaceae family. It has slender, gray-green leaves and bluish-purple flowers [21]. Previous phytochemical investigations showed that lavender essential oils are rich in linalool, linalyl acetate, and camphor. Prior research indicated that the antimicrobial effects of lavender oils are mainly related to their bioactive compounds [16, 22].

Previous investigations have reported the antibacterial effects of the three studied EOs. Indeed, it has been reported that the OMEO and its major constituents, such as *p-*menthane, germacrene D, and *α*-pinene, exhibit powerful antibacterial properties against various strains, especially *Escherichia coli* and *Staphylococcus aureus* [20, 23–25]. Numerous studies reported the antimicrobial properties of the species belonging to the genus *Lavandula* and *Eucalyptus,* mainly *Lavandula stoechas, Lavandula intermedia,* and *Eucalyptus globulus* against a plethora of microbial strains [26–28].

The present investigation aims to analyze the phytochemistry of the EOs extracted from *Eucalyptus polybractea cryptonifera* (EPEO), *Ormenis mixta* (OMEO), and *Lavandula burnatii briquette* (LBEO) and evaluate their single antimicrobial activity as well as to determine the combination that affords the optimal inhibitory ability against the tested bacterial strains. Consequently, the simplex-centroid mixture design was employed to create polynomial models elucidating the relationship between the antibacterial effect and the amount of each volatile oil. *Eucalyptus polybractea cryptonifera*, *Ormenis mixta*, and *Lavandula burnatii briquette* were selected based on their medicinal applications. To the best of our knowledge, the essential oils derived from these plants have not been the subject of previous investigations. Hence, this is the first research aiming to combine these plants' essential oils and elucidate their antimicrobial and phytochemical characteristics.

The following points represent the hypothesis of this study:

- The phytochemical analysis of the studied EOs will reveal the presence of diverse chemical compounds that could be responsible for the antimicrobial properties of these EOs
- Every single EO will exhibit important antimicrobial activity against the tested bacterial strains, demonstrating their potential as natural antibacterial agents
- The optimal mixtures of EPEO, OMEO, and LBEO resulting from the simplex-centroid mixture design will exhibit enhanced antibacterial effects compared to the single EOs
- The polynomial models will elucidate the quantitative relationship between the amount of each oil in the mixture and its corresponding antibacterial effect, providing valuable insights into the synergistic interactions of the studied EOs

## 2. Materials and Methods

### 2.1. Plant Material and EOs Extraction

The aerial parts (stems, leaves, and flowers) of *Eucalyptus polybractea cryptonifera*, *Ormenis mixta*, and *Lavandula burnatii briquet* were harvested during the period between March and April at the flowering stage, since this is the best time to use plant essences more effectively [29].

The three plants were collected in the region of Taounate, Morocco (34° 32′ 09″ N, 4° 38′ 24″ W). Plant authenticity was carried out by the botanists of the Sidi Mohamed Ben Abdellah University, Fez, Morocco, and deposited under voucher codes of BLMUP 386-388.

The samples were dried to a constant weight for 10 days at 25°C in a dark place, crushed employing an electric blender, and sieved to attain a fine powder. Extraction of EOs from the plant aerial parts was executed by the hydrodistillation technique using Clevenger-type device. This technique features a recycling system and operates at atmospheric pressure. The system enables the preservation of mass plant/water proportion at its initial level. Through each extraction, 500 g of the dried aerial part of each plant was placed in a 1 L flask, and then 800 ml of distilled water was added. The solution was heated to boiling temperature (100°C) during 3 h; the released steams traversed the column and passed out of the condenser in a liquid form (extraction was performed in three separate replicates (*n* = 3)). At the end of the distillation process, two phases were noticed, an organic phase (EOs) and an aqueous phase (aromatic water). The attained oils were desiccated by anhydrous sodium sulfate and kept at 4°C pending upcoming tests.

### 2.2. Characterization of EOs Components

The analysis of *E. polybractea* (EPEO), *O. mixta* (OMEO), and *L. burnatii* (LBEO) was executed by gas chromatography coupled with mass spectrometry (GC/MS) and gas chromatography coupled with flame-ionization detector (GC-FID).

#### 2.2.1. GC-FID Analysis

Analytical GC was performed using a Hewlett-Packard (HP/Agilent 6890) device equipped with a FID apparatus. The separation was accomplished using an HP-5 MS no-polar capillary column (length 30 m, diameter 0.25 mm, film thickness 0.25 *μ*m).

The column temperature was set from 50°C to 200°C at 4°C/min. The chromatography carrier gas (nitrogen) was fixed to 1.4 ml/min, and split injection mode was used with a 1/50 split ratio; the temperature of injector and detectors (FID) was set at 250°C. The volume of oils (diluted 1/20 v/v in methanol) injected was approximately 1 *μ*l. The device was controlled by an “HP Chem station” computer system, which managed its operation and allowed the monitoring of chromatographic analyses.

#### 2.2.2. GC-MS Analysis

The GC-MS analysis was executed with a Hewlett-Packard gas chromatography (HP6890) equipped with a mass spectrometry system (HP 5973). Chromatographic separations were carried out using an HP-5 MS capillary column (30 m × 0.25 mm i.d., 0.25 *μ*m film thickness). The carrier gas was helium whose flow rate was fixed at 1.4 ml/min.

The column temperature was managed at 50 and 200°C with a rate of 4°C/min. The injection of 1 *μ*L of EOs (diluted 1/20 in methanol solution) was performed, split ratio 1 : 30. The mass spectra (MS) were programmed over a scan range of 30 to 1000 amu; 0.5 s/scan. The ionization energy was 70 eV. The temperature at the ionization source and the input was 280°C.

The volatile compounds were identified based on their retention indices (RIs) and MS, which were compared with those obtained in the literature [30, 31]. Moreover, the mass spectra (MS) of various compounds were verified using standardized data from chemical libraries, including the NIST 2022 and the Wiley 275. Finally, commercialized standards (terpenes with purities ranged between 80 and 98%) were also used for external standardization.

### 2.3. Antibacterial Assays

Prior to formulation, the antibacterial activities of *E. polybractea*, *O. mixta,* and *L. burnatii* EOs were investigated separately. In order to assess the EOs antibacterial activity, the first step was the use of the disc diffusion method which allowed identifying the concentration giving a response classified as sensitive. Second, the minimum inhibitory concentration (MIC) and minimum bactericidal concentration (MBC) were determined for each EO.

#### 2.3.1. Microorganisms

The EOs were examined against two reference bacterial strains: *Escherichia coli* ATCC 25922 and *Staphylococcus aureus* ATCC 29213. Both strains were obtained from the laboratory of Microbial Biotechnology and Bioactive Molecules, Science, and Technology Faculty, Fez. Before being used, the strains were reactivated by subculturing in Luria–Bertani (LB) plates at 37°C for 18–24 h.

#### 2.3.2. Disc Diffusion Method

The antimicrobial activity of the studied EOs was determined using the agar disc diffusion method with minor reforms [32]. This method has been used as a first step to assess the inhibition diameters generated by the EOs around the disk.

A fresh culture suspension was prepared in sterile saline solution and adjusted to 0.5 McFarland (10^8^ CFU/mL), then inoculated into Mueller Hinton Agar (MHA) plates and incubated briefly for 20 min, and the culture's excess was eliminated. The sterile paper discs (6 mm diameter) were soaked with 5 *μ*l of pure EO from each plant before being put on an inoculated agar plate. In addition, gentamicin (50 *μ*g/disc) and kanamycin (50 *μ*g/disc) were employed as standards to identify the sensitivity of the tested strains, while DMSO (5 *μ*L; 5%) was used as growth control. These plates were maintained at 4°C for 3 h and then incubated at 37°C for 24 h. After incubation, the inhibition zone diameters were measured in millimeters using a digital Vernier caliper (Mitutoyo). The results were represented as the mean ± standard deviation for three separate tests (*n* = 3).

#### 2.3.3. Determination of MIC and MBC

MICs of EPEO, OMEO, and LBEO were assessed using the broth microdilution assay in 96-well microplates as previously explained [32, 33] with minor adjustments. Bacteriological agar was employed as an emulsifier of EOs in a culture medium at 0.15% (v/v), and p-iodo-nitrotetrazolium chloride (INT) 95% (Sigma-Aldrich) was used as a bacterial growth indicator. First, a microtiter plate was filled from the second to the twelfth well, with 50 *μ*l of Mueller–Hinton broth (MHB) supplemented with agar (0.15%). Then, 100 *μ*L of EOs dilution prepared in MHB with agar (0.15%) was added to the first test well of each microtiter row. Next, 50 *μ*l of scalar dilution was moved from the second to the eleventh. The 12^th^ well was considered as growth control. Then, 50 *μ*L of the bacterial suspension prepared and adjusted to 0.5 McFarland (10^8^ CFU/mL) was deposited in each well.

The plates were incubated at 37°C for one day (24 h). Then, 5 *μ*l of INT was added to each well. After 2 hours of incubation, the MIC was defined as the maximum EOs dilution where the white-to-red color shift was unnoticeable. Tests were conducted in triplicate (*n* = 3). MBC was evaluated by subculturing 15 *μ*L from each negative well on LB agar plates and incubating for 18–22 h at 37°C. MBC was the lowest concentration at which no growth was detected. Moreover, the MBC/MIC ratios were also determined to identify the possible mechanism of the studied EOs [34].

### 2.4. Synergism Testing

The checkerboard technique was performed to verify synergistic interactions between the tested EOs against bacterial strains [35].

The tested sample concentrations were prepared in MHB containing 0.15% bacteriological agar. On the microplate *x*-axis, 25 *μ*L of the weakest active EO concentration, which corresponds to the highest MIC value of EO (determined by microdilution assay) was added to the well from the 1^th^ to the 11^th^ one. Regarding the *y*-axis, 25 *μ*L of each higher active EO concentration, which consists of the lowest MIC value of EO (determined by microdilution assay) was added to each well. The 12^th^ well served as a negative control. Then, 50 *μ*L of the bacterial suspensions, at a concentration of 2 × 10^6^ UFC/mL, was added to each well.

Thereafter, the microplate was incubated at 37°C for 18 to 20 h. After that, 10 *μ*L of resazurin was added to each well as a bacterial growth indicator. After incubation at 37°C for 90 min, bacterial growth was detected by reduction of blue dye (resazurin) to pink (resorufin). Experiments were perfomed in triplicate (*n* = 3).

The synergy was evaluated based on the instructions described by the American Society for Microbiology [36].

The fractional inhibitory concentration (FIC) index values were determined as follows:

(1) $$\begin{array}{c} \sum FICI=FIC\left(E_{A}\right)+FIC\left(E_{B}\right)\text{,} \end{array}$$

with

(2) $$\begin{array}{c} FIC\left(X_{A}\right)=\frac{MIC\left(E_{Acombination}\right)}{MIC\left(E_{Aalone}\right)}\text{,}\\ FIC\left(X_{B}\right)=\frac{MIC\left(E_{B\;combination}\right)}{MIC\left(E_{Balone}\right)}\text{.} \end{array}$$

The ∑FICI index was interpreted as follows: synergistic = FIC ≤0.5; partial synergy = 0.5 < FIC ≤ 0.75; additive interaction = 0.76 ≤ FIC ≤ 1.0; indifferent (noninteractive) = 1.0 < FIC ≤ 4.0; and antagonistic interaction = FIC >4.0.

### 2.5. Experimental Design

#### 2.5.1. Mixture Design

The optimization and evaluation of antibacterial activity were generated using an augmented simplex-centroid design for three compounds [37]. For this, ten trials were conducted, using three pure EOs at the triangle's vertices (*E*_1_ − *E*_2_ − *E*_3_), binary mixtures at the three triangle's sides (*E*_4_ − *E*_5_ − *E*_6_), equiproportional mixture of the EOs at the triangle's centroids (central point) (*E*_7_), and control points (*E*_8_ − *E*_9_ − *E*_10_).

**Figure 1:**
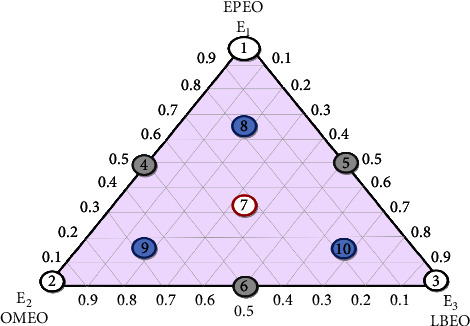
An overview of the simplex-centroid design for three-compound mixtures.

The experiment of the equal proportionate mixtures has been tripled to determine the lack of the model fit. Ultimately, 12^th^ experiments were used in the current exploratory design (Figure 1).

Factors (*E*_1_, *E*_2_, and *E*_3_) are used to explain response variation in a mixture, and they represent a portion of each experimental component in the mixture, which has a value range between 0 and 1 without constraints [38], with

 
*E*_1_: the proportion of EOEO
 
*E*_2_: the proportion of OMEO
 
*E*_3_: the proportion of LBEO

The responses assessed in this investigation were the antibacterial action against two bacteria, including *S. aureus* and *E. coli*. Next, the data were fitted to a special cubic model employing least-squares regression to reveal the unidentified coefficients in the following equation [39]:

(3) $$\begin{array}{c} Y={\delta_{1}E}_{1}+{\delta_{2}E}_{2}+{\delta_{3}E}_{3}+\delta_{12}E_{1}E_{2}+\delta_{13}E_{1}E_{3}+\delta_{23}E_{2}E_{3}+\delta_{123}E_{1}E_{2}E_{3}+ɛ\text{,} \end{array}$$

where *Y* is the MIC response in % (v/v). *δ*_1_, *δ*_2_, and *δ*_3_ are the coefficients of linear terms. *δ*_12_, *δ*_23_, and *δ*_23_ are the coefficients of binary terms. *δ*_123_ is the coefficient of ternary term. *ɛ* is the error term.

#### 2.5.2. Statistical Analysis

The *F*_LOF/PE_ ratio between the mean square lack of fit (MS_LOF_) and the mean square pure error (MS_PE_) was used to ensure the accuracy of the model with observations. High *F*_LOF/PE_ values signify a mismatch to the model [40, 41]. Moreover, the validation of the fitted models was verified using the ANOVA *F*-test. To determine the statistical significance of the model, we used the ratio between the mean square regression (MS_*R*_) and the mean square residual (MS_*r*_) [42]. Then, the quality of the assumed model was further assessed using the coefficient of determination *R*^2^. In fact, it is frequently presented as a percentage (%) and used to assess the correlation among observed and expected responses [43], whereas Student's *t*-test was employed to determine the importance of the model's coefficients [44]. This analysis was executed applying JMP software version 14 and Design Expert version 12.

#### 2.5.3. Optimization Tools

The contour plot and 3D surface, based on iso-response curves, were employed to identify the ideal EOs formulation, resulting in the required responses. These curves were used to look for the factors modification intervals to get the desired response [43, 45].

Besides, the desirability test was applied to identify the desired response values based on the optimal conditions. Thanks to this tool, we can provide the precise optimum setting with a percentage between 0 and 1. A value of 1 is given when factors result in the maximum desired response, whereas a value of 0 signifies an inadmissible response [46].

## 3. Results and Discussion

### 3.1. Chemical Composition

The EOs yields (v/w) were 2.13%, 0.9%, and 1.38% for *E. polybractea*, *O. mixta,* and *L. burnatii*, respectively.

EOs generally include compounds derived from two major groups: monoterpenes and sesquiterpenes (hydrocarbons and their derivatives) [47]. Monoterpenes are a chemical family of terpenes that possess two isoprene units per component with a structural formula of C_10_H_16_ (monoterpenes hydrocarbons). Monoterpenes may be linear (acyclic) or comprise a single (monocyclic) or double rings (bicyclic) [48]. Modified terpenes, such as oxygen-containing components are known as oxygenated monoterpenes. Furthermore, sesquiterpenes are secondary metabolites consisting of 15-carbon components containing three isoprenoid units and representing a multifaceted and heterogeneous subclass of bioactive molecules [47, 49].

The chemical analyses of EPEO, OMEO, and LBEO, including the percentage of each constituent, elution order, molecular formula, and retention index, are summarized in Table 1. A total of 70 volatile components have been detected in the three studied EOs. Indeed, twenty-five, twenty-nine, and thirty-three components were identified in EPEO, OMEO, and LBEO, representing 98%, 94.82%, and 97.47% of these oils, respectively.

EPEO was characterized by a high amount of monoterpene hydrocarbons (57.18%) and a low amount of sesquiterpene hydrocarbons (0.97%). Besides, LBEO has revealed an important percentage of oxygenated monoterpenes (62.49%) and a moderate proportion of oxygenated sesquiterpenes (0.49%). As regards, OMEO, sesquiterpene hydrocarbons, and oxygenated monoterpenes were the most dominant constituents with 36.27% and 27.67%, respectively.

The chemical analysis of EOs attained from the three studied plants revealed that 1,8-cineole (23.75%), *p*-cymene (22.47%), and *α*-pinene (11.20%) were the main bioactive compounds detected in the EPEO. These findings are in agreement with those cited by prior investigations mostly for 1,8-cineole dominance [50–53]. However, many studies that focused on the chemical composition of the genus *Eucalyptus* (*E. dives*) EO revealed other chemotypes: piperitone (40.50%), *α*-phellandrene (17.40%), and *p*-cymene (8.50%) with a low rate of 1,8-cineole (0.70%) [54].

This difference may be ascribed to several factors, including environmental conditions (soil type, precipitation, and climate), plant origin, harvesting time, extraction and processing methods, and phenological stage of plant concerned, and it could be genetically determined [41, 55, 56].

As regards the analyzed OMEO, twenty-nine compounds were identified, with p-menthane-p1,8-diol (18.19%), *α*-pinene (10.81%), and D-germacrene (9.17%) as major compounds. Prior investigations have determined the chemical composition of OMEO from other Moroccan areas and have revealed varying volatile constituents that depend on the plants' origin. For instance, the oil of *O. mixta* collected in the Taounate region mainly contains santolina alcohol, farnesene, and epi-*α*-macrogol [8]. Furthermore, Elouaddari et al. [57] found that the chemical composition of OMEO from Morocco varies qualitatively and quantitatively based on geographical location and growth conditions. Indeed, the OMEO collected from Benguerir, Kenitra, Settat, Meknes, and Tamesna regions contained camphor and *β*-myrcene, whereas the *β*-myrcene and *β*-farnesene chemotypes were present in the Chefchaouane's sample. However, the Bouznika sample contained methacrylate and 2-methyl-2-trans-buteny [58].

Concerning the phytochemical profile of LBEO, thirty-three constituents were identified, of which linalool was the major component with a percentage of 24.40%, followed by linalyl acetate (18.68%) and camphor (8.01%). According to Lesage-Meessen et al. [59], these compounds are primarily in charge of lavandin distinctive flavor as well as its biological and therapeutic characteristics. This outcome is in concordance with those described in the literature. Indeed, for the lavandin EO from Iran, 1,8-cineole was the major component (47.90%) followed by borneol (26.40%) and camphor (14.40%) [60]. Moreover, *L. angustifolia* EO from Italian origin (Salerno) was characterized by a high content of linalool (33.10%) and linalyl acetate (10.40%) [61]. Furthermore, the main constituents in lavandin (*Lavandula* × *intermedia*) grown in Lazio region (Italy) were also linalool (41.60%) and linalyl acetate (23%), with smaller amounts of 1,8-cineole (5.20%) [62]. In contrast, Romanian lavandin EO was richer in camphor and eucalyptol [26]. The fluctuations in the percentages of detected compounds may be related to either the altitude of the cultivation area and/or its specific microclimate conditions [27, 63–65].

### 3.2. Single Antibacterial Activity

**Figure 2:**
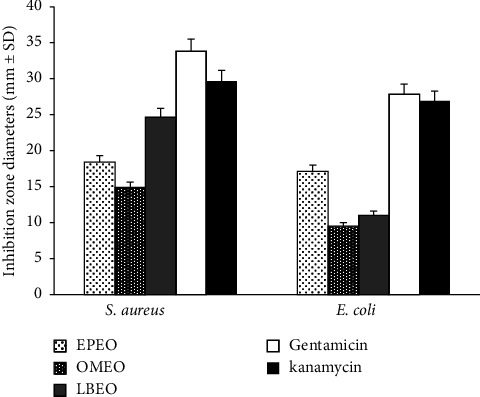
Screening of antibacterial action by disc diffusion method of EPEO, LBEO, and OMEO and standard antibiotics (gentamicin and kanamycin) against *S. aureus* and *E. coli.*

Results of the antibacterial activity of EPEO, OMEO, and LBEO examined by disc diffusion method against two bacterial strains are summarized in Figure 2.

The inhibition zone diameter values are depending on the tested EOs' nature and the tested species' susceptibility. LBEO, EPEO, and OMEO showed an important antibacterial effect against *S. aureus* with respective inhibition zone diameters of 24.66 ± 1.45, 18.4 ± 0.655, and 14.9 ± 0.75 mm. Moreover, this noticeable antibacterial effect has been also revealed against *E. coli* with 17.13 ± 2.85 mm, 11.03 ± 1.05 mm, and 8.52 ± 0.82 mm for EPEO, LBEO, and OMEO, respectively. These effects are less effective compared with the standard antibiotics (gentamicin and kanamycin), which may be explained by the fact that antibiotics are particular antimicrobial drugs with specific site of action on bacteria from the one hand [66]. On the other hand, EOs consist of a large number of bioactive compounds, which may lead to antagonistic interactions, thus limiting the antimicrobial activities of EOs.

The quantitative antibacterial values (MIC and MBC) of the three tested EOs against *S. aureus* and *E. coli* are presented in Table 2.

All studied EOs have presented significant antibacterial activity. In fact, *S. aureus* appears to be the most sensitive strain to the three EPEO, LBEO, and OMEO, with MIC values ranging from 0.25 to 0.5% (v/v). Indeed, LBEO and EPEO have the strongest antibacterial activity with MIC values of 0.25% (v/v). Moreover, noticeable antibacterial effect has also been revealed for OMEO with MIC equal to 0.5% (v/v).

However, a lower antibacterial effect has been shown against *E. coli* with MIC values ranging from 1 to 6% (v/v). In fact, LBEO exhibited the highest antibacterial activity (1% v/v) followed by EPEO (2% v/v), while OMEO showed the weakest antibacterial effect (6% v/v). This is to note that the standard antibiotics gentamicin and kanamycin have shown MIC values ranging from 8 to 32% (v/v) against both strains. The MBC values of the three tested EOs are quite similar to the MIC values obtained against *E. coli* and *S. aureus*. However, for EPEO, the MBC value was twice as high as the MIC. Concerning this bactericidal effect, it has been found that the Gram-positive (Gram+) bacterial strain is more sensitive to EPEO, LBEO, and OMEO than the Gram-negative (Gram−) bacteria.

The antibacterial activity of EOs could be elucidated by the molecular interaction of the functional groups of their components with the bacterial cell wall, causing whole-cell lysis.

Liberation of internal components from the cell is a good indicator of membrane integrity, with small ions such as phosphate and potassium that have a tendency to diffuse before large molecules such as RNA and DNA and other substances [28, 67]. Moreover, the resistance of Gram-negative (Gram^−^) bacteria is strongly linked to the composition of their cell wall which limits the diffusion of hydrophobic components such as EOs and their bioactive components through the lipopolysaccharide (LPS) layer [68, 69]. However, LPS are absent in Gram-positive bacteria, which possess a cell wall mainly constituted by a peptidoglycan layer facilitating the diffusion of EOs through cell membrane and thereby distributing cell permeability and binding with vital macromolecules, such as proteins, DNA, and RNA and thus causing cell death [66].

Previous investigations have reported the antibacterial effects of the three studied EOs. Our findings are consistent with those reported by Ouedrhiri et al. [20] who showed that OMEO causes a significant inhibition on Gram^+^ bacteria (*S. aureus* and *B. subtilis*), while a weak antibacterial effect was determined against Gram^−^ bacteria, including *Pseudomonas aeruginosa* and *E. coli* [8, 20].

As it can be observed, the Gram^−^ strains are more resilient than the Gram^+^ ones. Indeed, the Gram^−^ have an envelope that consists of three layers. The first layer is the outer membrane, a protective and a unique feature that distinguishes Gram^−^ from Gram^+^ bacteria [3]. The outer membrane of Gram^−^ strains is the principal reason for resistance to a broad variety of antimicrobial agents including essential oils due to their hydrophobic characteristics [10]. Besides, the Gram^−^ strains can change their hydrophobic properties via mutations, creating resistance to EOs, while the Gram^+^ ones do not have this strong layer, which makes Gram^−^ bacteria more resistant than Gram^+^ ones [70].

Nevertheless, it has been previously shown that the major constituents of OMEO (*p-*menthane, germacrene D, and *α*-pinene) exhibit antibacterial effect against *E. coli* and *S. aureus* [20, 23–25]. Therefore, the lower antibacterial action found against Gram^−^ bacteria might account for an antagonistic interaction among its volatile compounds. Indeed, numerous researches have shown that the antibacterial effect of EOs is controlled by the intricate interplay between their minor and major constituents. In some cases, these components are active against bacterial cells when evaluated separately [20, 71].

To the best of our knowledge, there is no literature data demonstrating the antibacterial activity of LBEO. However, numerous studies reported the antibacterial activity of the genus *Lavandula*. Indeed, Bouyahya et al. [72] showed that *Lavandula stoechas* exhibits antibacterial activity with some variability depending on the tested bacteria, experimental methods used, and/or chemical composition of the EOs. In addition, similar findings have been reported by Dadaliogÿlu and Evrendilek and Cherrat et al. [73, 74].

Furthermore, Garzoli et al. [62] showed that lavandin (*Lavandula intermedia*) EO exhibits bactericidal activity against a wide range of pathogenic bacteria. This effect can be attributed mainly to the dominant presence of linalool (41.60%) in this oil. Indeed, Silva et al. [75] also attributed *Ocimum basilicum* EO activity to linalool. Furthermore, Hussain et al. [76] have demonstrated that *Ocimum basilicum* EO and linalool compound display antibacterial and antifungal actions against *E. coli*, *S. aureus*, *B. subtilis*, and *Aspergillus niger*. The research suggests that the antimicrobial activity of the LBEO could be associated with its high content of oxygenated monoterpenes which are highly effective against microbial cells [77].

In addition, EPEO has not gained much attention regarding its antibacterial properties. In this context, we have investigated this study to report the antibacterial activity of this plant. Djenane et al. and Oyedeji et al. [78, 79] obtained similar results with some variability against a panel of bacteria. Our EO exhibited significant bacteriostatic and bactericidal effects against *E. coli* and *S. aureus* compared to the results obtained by Fahad et al. [50], while these findings are corroborated with those reported by Assaggaf et al. [80], which indicated a strong antibacterial effect at low MIC and MBC values. These differences may be attributable to the variations in the EOs' chemical composition and variations in the experimental conditions as well as to bacterial strains tested.

The mechanism of EPEO, LBEO, and OMEO remains unresolved. Nevertheless, it has been found that other EOs have many antibacterial mechanisms [68, 81, 82]. Indeed, the mechanisms of action include the capacity to cross the cell membrane, the disturbance in the electron respiratory chain, and the leakage of electrolytes [81]. Other mechanisms have shown that EO components inhibit the quorum sensing signaling pathways, thus decreasing the bacterial resistance [83–85].

Besides, the antimicrobial properties of EOs can be attributed to the composition, functional groups of the bioactive compounds, and their synergistic interactions. Generally, terpenes and terpenoids are the main groups of EOs. They are characterized by a small molecular weight. The terpenoids group can be partitioned into alcohols, ketones, esters, phenols, aldehydes, epoxides, and ethers [81]. Many terpenoid compounds have demonstrated significant antimicrobial effects against various Gram^+^ and Gram^−^ strains, especially thymol, geraniol, carvacrol, linalyl acetate, menthol, piperitone, and linalool which are the major compounds of terpenoids. These compounds are able to interact with membrane proteins and disrupt the outer and inner membrane of bacteria, resulting in bacterial death [70, 80].

### 3.3. Optimization of Antibacterial Action by Mixture Design

#### 3.3.1. Antibacterial Formulations Design

Table 3 displays the mixtures design, which comprises different mixtures of OMEO, LBEO, and EPEO, along with the observed responses for each experiment on *S. aureus* and *E. coli*. The experiments were conducted randomly and each response is the mean of three repetitions. The results demonstrated that the equiproportional mixture of three EOs and the ternary mixture (0.167: OPEO/0.167: OMEO/0.667: LBEO) were the most performant formulations, presenting the highest antibacterial activity against the two studied strains.

#### 3.3.2. Statistical Validation of the Postulated Model

The experimental response data were statistically analyzed to confirm the chosen model for each bacterial strain, which represents the relationship between responses and factors.

The variance analysis reveals that the regression main impact is statistically significant for the two examined responses since their risk significance (*p* value) is less than 0.05 (0.0001^*∗*^ and 0.001^*∗*^ for *E. coli* and *S. aureus*, respectively). In addition, the *F*_ratio(*R*/*r*)_ calculated for both studied responses are higher than the theoretical value at the 95% confidence level. As shown in Table 4, the *F*_ratio(*R*/*r*)_ for *E. coli* (117.694) and *S. aureus* (29.41) exceeded the tabular value of *F* at a 95% confidence level. Besides, the ANOVA *F*-test indicated that the both postulated models had no lack of adjustment, because their *p* values were higher than 0.05 (0.07 and 0.051). The computed *F*_Ratio(LOF/PE)_ of the investigated responses was also observed to be lower than the theoretical value *F*_(0.05; 3.2)_=19.16 at the 95% confidence level.

**Figure 3:**
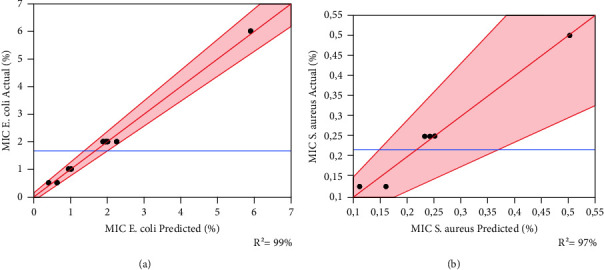
Curves of the observed values according to the predicted values for the two studied responses (a) *E. coli* and (b) *S. aureus.* The red lines show the curve of actual values of MIC as a function of those predicted for both tested strains. The blue horizontal lines indicate the mean of the observed values.

The coefficients of determination *R*^2^ for *S. aureus* and *E. coli* are 97% and 99%, respectively. These values indicate a sufficient agreement between the experimental values and those predicted by the fitted model. These findings were supported by the graph in Figure 3, which shows that the curves of observed values as a function of predicted values look exactly like a straight line.

#### 3.3.3. Factors Effects and the Fitted Model of Both Responses

The impact of all investigated factors, their corresponding *t-*student statistical values, and the *p* values, are summarized in Table 5. The mathematical model coefficients were statistically significant when their *p* values were lower than 0.05, while those with a *p* value higher than 0.05 were excluded from the presumed model.

The statistically significant coefficients for the MIC response of *E. coli* are linear terms (*δ*_1_, *δ*_2_, and *δ*_3_), binary interaction terms between EPEO and OMEO (*δ*_12_) as well as OMEO and LBEO (*α*_23_) and finally ternary term (*δ*_123_). These outcomes showed that the antibacterial action on *E. coli* is dependent on all terms in the modulated mathematical model, except for the coefficient associated with the binary term (*α*_13_) between EPEO and LBEO.

The mathematical model was estimated according to the following formula:

(4) $$\begin{array}{c} Y={2.02E}_{1}+{5.92E}_{2}+{0.96E}_{3}-8.12E_{1}E_{2}-6.21E_{2}E_{3}-32.04E_{1}E_{2}E_{3}+ɛ\text{.} \end{array}$$

Regarding the response MIC_*S.aureus*_, all the terms in the adapted mathematical model (*δ*_1_, *δ*_2_, *δ*_3_, *δ*_12_, *δ*_13_, and *δ*_123_) are statistically significant, except for the coefficient representing the binary mixture of EPEO and LBEO (*α*_13_). These findings reflect that the antibacterial effect against this bacterial strain depends on all interactions except those obtained by EPEO^*∗*^ LBEO.

The predictive mathematical mode was determined as follows:

(5) $$\begin{array}{c} Y={0.24E}_{1}+{0.5E}_{2}+{0.24E}_{3}-0.51E_{1}E_{2}-0.51E_{2}E_{3}-2.7E_{1}E_{2}E_{3}+ɛ\text{.} \end{array}$$

#### 3.3.4. Formulation Optimization and Desirability Study

The optimization process adopting the mixture design approach enables us to determine the optimal formulation of the three EOs demonstrating the best MIC value, that is to say, the smallest concentration which exhibits the highest sensitivity. The smallest MIC values noticed during the experiments were 0.5% and 0.125% for *E. coli* and *S. aureus*, respectively. Consequently, a formulation of the three EOs allowing to obtain responses smaller than or equal to these values will be elucidated.

**Figure 4:**
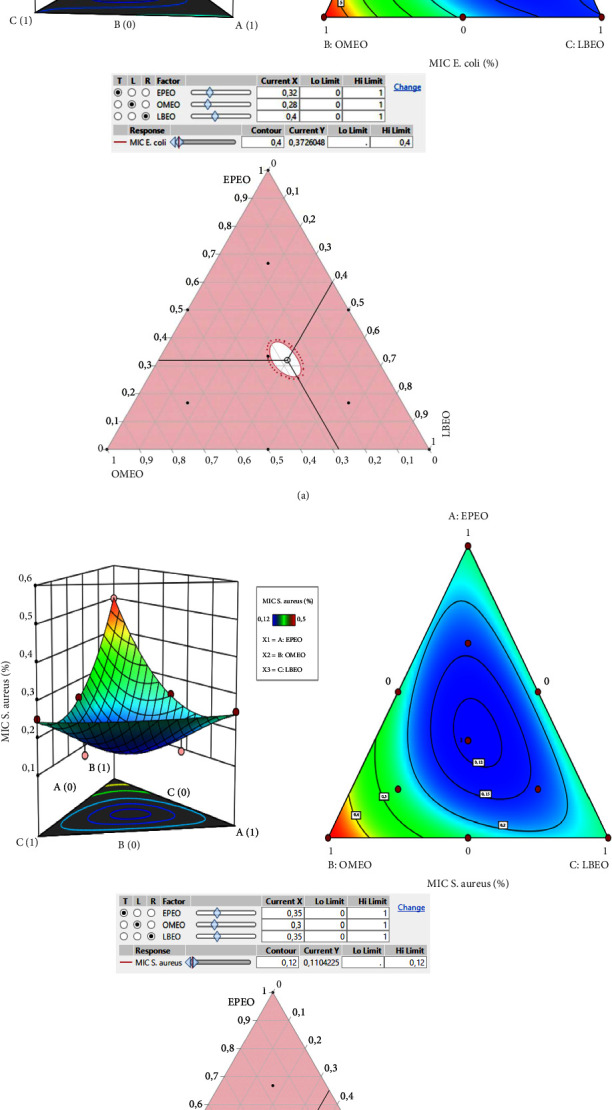
2D and 3D mixture plot displaying the optimal compromise area leading to the desired MIC values against *E. coli* (a) and *S. aureus* (b).

In the present research, it is important to emphasize that the ternary mixture indicates stronger antibacterial activity against both bacteria than single oils and binary mixtures. This positive combination is depicted in Figure 4, where the optimum mixture zone is situated in the center of the triangle. The contour plot and 3D surface (Figure 4) demonstrate the interaction among each component of the mixtures. This graph illustrates changes in response, with the dark blue zone representing weak MIC values and greater bacterial potential. However, the green to orange hue indicates elevated MIC values. Hence, the mixture design approach optimized the amounts of individual active constituents to generate an optimal formulation established by its potent antibacterial activity.

**Figure 5:**
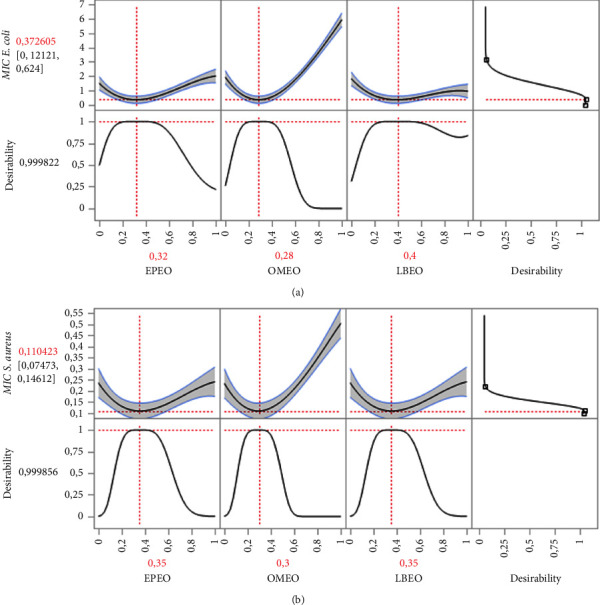
Desirability plot revealing the precise proportions of LBEO, OMEO, and EPEO leading to the best antibacterial action against *E. coli* (a) and *S. aureus* (b).

- Effect of the EOs formulation against *E. coli*
- The MIC_*E. coli*_ response achieved in the various tests ranged from 0.5 to 6% (Table 3).  Figure 4(a) indicates the contour and surface plots of the MIC_*E. coli*_ response found with diverse mixtures of the EPEO, OMEO, and LBEO. A MIC value equal to 0.40% was determined as a compromise against *E. coli*. From the 2D and 3D mixing graph, we can conclude that a mixture of EPEO, OMEO, and LBEO, is necessary to achieve this MIC value. In addition, the desirability function (Figure 5(a)) exhibited the precise amounts of EPEO, OMEO, and LBEO leading to the desired MIC value of 0.37%. Thus, the desirability test revealed that there is a 99.9% chance of reaching this value using the following mixture: 32%, 28%, and 40% of EPEO, OMEO, and LBEO, respectively.
- Effect of the EOs formulation against *S. aureus*
- The results of the microdilution assay indicated that the MIC_*S.aureus*_ response varied between 0.125 and 0.5% (Table 3). Moreover, 2D and 3D mixture plots (Figure 4(b)) illustrated the optimal compromise zone, revealing that the mixture comprising EPEO, OMEO, and LBEO is required to attain the desired MIC of about 0.12%.
- In fact, the desirability function (Figure 5(b)) confirms this finding, indicating that the ternary mixture with the following proportion (35 : 30 : 35 v/v/v) EPEO, OMEO, and LBEO leads to the best achievable MIC value (0.11%), with a compromise percentage of 99.9%. These outcomes demonstrate the synergistic interaction between these components.

Numerous investigators are currently using a mixture design methodology to assess the potential interactions between various mixture constituents in order to estimate the optimal formulation [38, 43, 86, 87].

Zieniuk and Bętkowska [86] employed the mixture design approach to evaluate and optimize the synergistic antibacterial effect among tea tree, rosewood, and lavender EOs against *E. coli*, *Listeria monocytogenes*, and *Rhodotorula mucilaginosa*. Within this line, the synergistic effects of *Eucalyptus camaldulensis*, *Mentha pulegium*, and *Rosmarinus officinalis* EOs against bacteria responsible for nosocomial infections were also investigated using a simplex-centroid design [38]. In addition, Fadil et al. [43] optimized the proportions of *Thymus. vulgaris* L., *R. officinalis* L., and *Myrtus communis* EOs by applying a centroid mixture design. The optimal formulation corresponded to 45% of myrtle and 55% of thyme EOs, which showed synergistic activity against *Salmonella typhimurium* strain.

#### 3.3.5. Interaction between EOs

**Figure 6:**
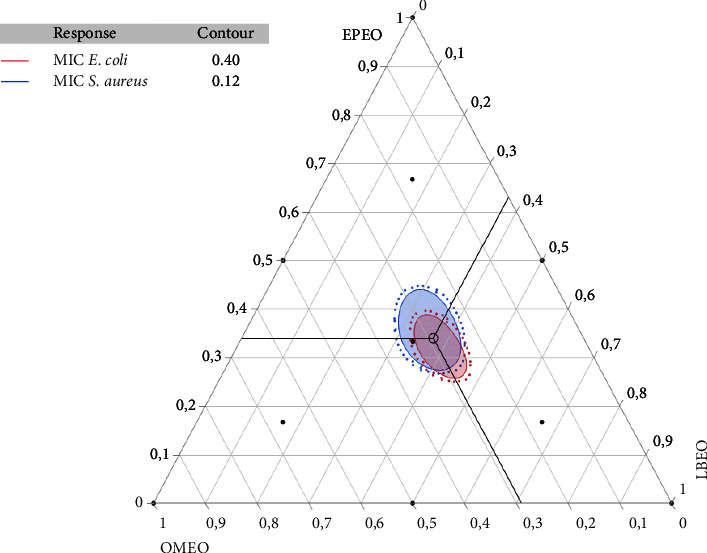
Mixture plot showing the optimal combination zone among *O. mixta*, *E. polybractea*, and *Lavandula briquit* against bacterial strain.

The mixture contour plot of *E. coli* and *S. aureus* responses, generated by the three EOs, EPEO, OMEO, and LBEO, elucidates the impact of the simultaneous optimization. The optimal compromise area for the two strains indicated that the desired MIC requires a ternary mixture of the abovementioned EOs (Figure 6).

The mixture of these components allowed particularly oxygenated monoterpenes (1,8-cineole, *p-*menthane, and linalool) and monoterpene hydrocarbons (*p*-cymene and *α*-pinene) to assemble. Each volatile compound has different sites within the bacterial cell where it can act [88]. The oxygenated terpenoids, including 1,8-cineole, *p*-menthane, and linalool, are the major antibacterial constituents as compared with the terpene hydrocarbons, which lack hydroxyl groups (-OH) [39, 89]. In addition, the combination of minor and/or major constituents may be responsible for the synergistic effect on bacteria [90]. *p*-Cymene and *α*-pinene are not effective antibacterials when acting alone, but their combination with oxygenated terpenoids such as linalool and/or 1,8-cineole has shown promising antibacterial activities. Therefore, *p-*cymene can swell bacterial cell membranes, facilitating the diffusion of linalool and 1,8-cineole into the cell membrane, leading to bacterial death.

Various synergistic antibacterial effects have been described for compounds or fractions of EOs when studied in binary/ternary mixtures [71, 91–95]. Ultee et al. [91] reported that cymene, combined with an oxygenated monoterpene (carvacrol), possess a synergistic activity against Gram^+^ bacteria. Moreover, the combination eugenol/linalool has shown a synergistic activity toward Gram^−^ bacteria [92]. Additionally, combinations of 1,8-cineole/limonene, 1,8-cineole/thymol, 1,8-cineole/*p*-cymene, and *α*-pinene/linalool have been found to exhibit synergistic antimicrobial activity [93, 94, 96].

In this respect, to our knowledge, the current study reports for the first time the synergistic activity of 1,8-cineole, p-Menthane-1,8-diol, and linalool. In addition, this combination could increase the levels of components with antimicrobial properties, such as D-limonene, *γ*-cadinene, and *α*-pinene [97, 98].

Taken together, these findings provide scientific evidence for potential applications of studied oils in combination to develop novel and effective antimicrobial agents, which may be useful in food packaging, food preservation, and elaboration of biopharmaceuticals. However, further investigations on the antibacterial action of these oils alone and/or in mixture are strongly required to clearly describe in detail how they could interact with each type of bacteria. Furthermore, in the literature, the results showed that the three EOs have effectively inhibited the growth of foodborne pathogens in vitro, whereas their action in food system model (*in vivo*) has not been reported. Indeed, more studies are needed in this subject to validate the possible applications of the three studied oils as natural additive in foods to persevere their microbiological security.

#### 3.3.6. Synergistic Activity Using Checkerboard Assay

FIC index findings of the binary combination between tested EOs on *E. coli* and *S. aureus* are summarized in Table 6. Three dual combinations were assessed, including EPEO/OMEO, EPEO/LBEO, and LBEO/OMEO.

FIC index of the binary combination between tested EOs ranged from 0.156 to 0.75. As regards *S. aureus*, it has been noticed that the combinations of 1/4 MIC_EPEO_ + 1/32 MIC_OMEO_, 1/8 MIC_EPEO_ + 1/16 MIC_OMEO_, 1/32 MIC_EPEO_ *+* 1/8 MIC_OMEO_, and 1/64 MIC_EPEO_ *+* 1/4 MIC_OMEO_ displayed synergistic effects with respective FIC indexes of 0.281, 0.1874, 0.156, and 0.265, whereas the combinations 1/2 MIC_EPEO_ + 1/4 MIC_OMEO_ and 1/256 MIC_EPEO_ + 1/2 MIC_OMEO_ exhibited a partial synergy with respective FIC indexes of 0.656 and 0.503.

Furthermore, 1/2048 MIC_EPEO_ + 1/4 MIC_LBEO_ showed a synergistic antibacterial activity on *S. aureus* with a FIC index of 0.25, while the combination of 1/4096 MIC_EPEO_ + 1/2 MIC_LBEO_ has shown a partial synergistic effect with a FIC index of 0.5002. Moreover, the combination of 1/4 MIC_LBEO_/1/2_OMEO_ also presented a partial synergistic activity, with a FIC index value of 0.75.

Concerning the effect of the tested EOs combination treatment against *E. coli*, it has been found that the combinations of 1/4 MIC_EPEO_ + 1/4 MIC_OMEO_ had an antibacterial synergistic activity with a FIC index of 0.5, whereas the combination 1/2 MIC_EPEO_ + 1/8 MIC_OMEO_ exhibited partial synergy with a FIC index of 0.625. The combinations of EPEO/LBEO and LBEO/OMEO demonstrated a synergistic and partial synergistic effect against *E. coli*.

The mixture of 1/4 MIC_EPEO_ + 1/512 MIC_LBEO_ and 1/2 MIC_LBEO_ + 1/4 MIC_OMEO_ displayed synergy with respective FIC index values of 0.251 and 0.5.

In contrast, four combinations showed partial synergy with a FIC index ranging from 0.50024 to 0.625. Each checkerboard assay produces diverse combinations. However, the FIC values of the most efficient combination are employed to assess the FIC index [71].

Taken together, the binary combinations between the three tested EOs had a greater antibacterial effect (synergistic) than the application of EOs alone, when tested against *E. coli* and *S. aureus.* It has been demonstrated that the minor compounds are involved in antibacterial effect and may exhibit synergistic interactions with other constituents [68].

The synergistic effect observed in this research between the studied EOs could be explained by the molecular interaction of the functional groups (monoterpene hydrocarbons, oxygenated monoterpenes, and sesquiterpene hydrocarbons). They integrate and disrupt the cell membrane and thereby facilitate the uptake of the active compounds, leading to cell lysis [99].

Other investigations also reported a synergic effect among *O. mixta*, *Eucalyptus*, and *Lavandula* EOs with other EOs [8, 20, 70, 100, 101]. In fact, Ouedrhiri et al. [20] highlighted the effect of the combination of *O. Mixta* and *Pelargonium asperum* EOs. The results showed that *O. mixta* MIC decreased from 2 to 0.007813% (v/v) against *S. aureus* after combination with *Pelargonium asperum* EOs. On the other hand, a synergistic activity was attained between *Eucalyptus* and *Dracocephalum* EOs against *S. aureus* and *E. coli* [70].

Furthermore, Moussii et al. [101] showed that the combination of lavender, wormwood, and rosemary EOs display a synergistic effect against Gram^+^ and Gram^−^ bacteria. A number of reports have proposed certain specific mode of action of antibacterial interaction that produce synergism outcomes, including modulating certain common biochemical pathways, inhibiting the protective enzymes, and using the active agents on the cell wall to increase the absorption of other antimicrobials [102]. In addition, volatile compounds derived from various medicinal plants possess hydroxyl functions (-OH) in their structure, which potentiate the antibacterial properties of terpenes [103].

Furthermore, the presence and position of functional groups in EO compounds may effectively modulate its antibacterial effect [66, 104]. In fact, in Gram^−^ bacteria, the presence of phenolic groups in carvacrol and thymol have been shown to interact with the outer membrane constituents, causing its breakdown and thereby leading to the liberation of LPS and increasing the membrane permeability with significant loss of ATP [105]. Moreover, some components such as carvone, which have a hydroxyl group (in position 3), appear to be responsible for its interaction with the bacterial wall, causing significant injury, especially in Gram+ bacteria [106]. Furthermore, it has been demonstrated that the antibacterial activity of terpene aldehydes is related to the electronegative characteristics of aldehyde group [107]. In fact, aldehydes can act on bacterial cell wall, restricting its biological functionality, especially electron transfer.

## 4. Conclusion

In this exploratory investigation, we reported the antibacterial formulation of three EOs derived from *Eucalyptus polybractea cryptonifera*, *Ormenis mixta*, and *Lavandula burnatii briquet* using checkerboard and mixture design approaches. The antibacterial action of these EOs depends on the proportion of each constituent and the target bacteria. As a result, it has been shown that the MIC values were considerably reduced using the combination of *E. polybractea*, *O. mixta*, and *L. burnatii.* These effects are mainly ascribed to the synergistic action of the major and/or minor molecules identified in the combined EOs. The most effective combinations on *E. coli* and *S. aureus* correspond to 32%, 28%, and 40% and 35%, 30%, and 35% of *E. polybractea*, *O. mixta*, and *L. briquet*, respectively. These antibacterial formulations may be suitable as alternative to commercialized antibacterial and preservative agents, which are increasingly becoming nonactive against a panel of bacterial strains causing serious infections and undesirable deteriorations of some food-based products.

## Figures and Tables

**Table 1:** Chemical composition of EPEO, OMEO, and LBEO.

No.^a^	Compounds^b^	Molecular formula	RI^c^	RI lit^d^	% of relative peak area			Identification
					EPEO	OMEO	LBEO	
1	*α*-Thujene	C_10_H_16_	902	905	0.94	—	—	MS, *I*_*R*_
2	Camphene	C_10_H_16_	943	943	0.26	—	—	MS, *I*_*R*_
3	*α*-Pinene	C_10_H_16_	948	948	**11.20**	**10.81**	0.33	MS, *I*_*R*_
4	3-Octanone	C_8_H_16_O	952	948	—	—	0.40	MS, *I*_*R*_
5	*β*-Myrcene	C_10_H_16_	958	958	0.72	—	—	MS, *I*_*R*_
6	*p*-Menthane-1,8-diol	C_10_H_20_O_2_	970	973	—	**18.19**	—	MS, *I*_*R*_
7	*β*-Pinene	C_10_H_16_	972	972	8.08	3.17	3.73	MS, *I*_*R*_
8	Sabinene	C_10_H_16_	975	976	—	2.29	—	MS, *I*_*R*_
9	Yomogi alcohol	C_10_H_18_O	998	999	—	1.96	—	MS, *I*_*R*_
10	*α*-Phellandrene	C_10_H_16_	1005	1002	1.76	—	—	MS, *I*_*R*_
11	D-Limonene	C_10_H_16_	1018	1020	—	—	1.10	MS, *I*_*R*_
12	Sabinene hydrate	C_10_H_18_O	1041	1040	—	—	1.92	MS, *I*_*R*_
13	p-Cymene	C_10_H_14_	1042	1041	**22.74**	—	0.33	MS, *I*_*R*_
14	*β*-Ocimene	C_10_H_16_	1051	1050	—	—	2.51	MS, *I*_*R*_
15	Terpinolene	C_10_H_16_	1052	1053	0.87	—	—	
16	1,8-cineole	C_10_H_18_O	1059	1059	**23.75**	—	6.57	MS, *I*_*R*_
17	*γ*-Terpinene	C_10_H_16_	1062	1060	7.29	0.97	—	MS, *I*_*R*_
18	Linalool oxide	C_10_H_18_O_2_	1080	1079	—	—	1.21	MS, *I*_*R*_
19	Linalool	C_10_H_18_O	1082	1082	0.62	—	**24.40**	MS, *I*_*R*_
20	Artemisia alcohol	C_10_H_18_O	1084	1083	—	1.95	—	MS, *I*_*R*_
21	1-Octenyl-3-acetate	C_10_H_18_O_2_	1109	1115	—	—	0.49	MS, *I*_*R*_
22	Camphor	C_10_H_16_O	1121	1122	—	—	**8.01**	MS, *I*_*R*_
23	(E)-Pinocarveol	C_10_H_16_O	1131	1132	0.59	1.17	—	MS, *I*_*R*_
24	Borneol	C_10_H_18_O	1138	1134	0.56	—	5.26	MS, *I*_*R*_
25	Fenchyl alcohol	C_10_H_18_O	1138	1137	0.48	—	—	MS, *I*_*R*_
26	*α*-Terpineol	C_10_H_18_O	1143	1144	3.11	—	1.71	MS, *I*_*R*_
27	Lavandulol	C_10_H_18_O	1146	1148	—	—	1.16	MS, *I*_*R*_
28	Allo-Ocimene	C_10_H_16_	1147	1147	0.66	—	0.61	MS, *I*_*R*_
29	Artemisyl acetate	C1_2_H_20_O_2_	1153	1152	—	1.75	—	MS, *I*_*R*_
30	Terpinen-4-ol	C_10_H_18_O	1177	1177	2.85	1.26	3.79	MS, *I*_*R*_
31	Hexyl butanoate	C_10_H_20_O_2_	1183	1182	—	—	0.84	MS, *I*_*R*_
32	Cryptone	C_9_H_14_O	1188	1188	2.69	—	—	MS, *I*_*R*_
33	[E]-3-Caren-2-ol	C_10_H_16_O	1188	1188	2.66	—	—	MS, *I*_*R*_
34	Cumin aldehyde	C_10_H_12_O	1230	1232	1.57	—	—	MS, *I*_*R*_
35	Carvacrol	C_10_H_14_O	1262	1269	0.70	—	—	MS, *I*_*R*_
36	Lavandulyl acetate	C_12_H_20_O_2_	1270	1271	—	—	2.98	MS, *I*_*R*_
37	Linalyl acetate	C_12_H_20_O_2_	1272	1270	—	—	**18.68**	MS, *I*_*R*_
38	Bornyl acetate	C_12_H_20_O_2_	1277	1273	—	1.39	—	MS, *I*_*R*_
39	Cuminol	C_10_H_14_O	1284	1283	0.42	—	—	MS, *I*_*R*_
40	Linalyl propionate	C_13_H_22_O_2_	1319	1318	—	—	0.55	MS, *I*_*R*_
41	Hexyl tiglate	C_11_H_20_O_2_	1331	1331	—	—	0.48	MS, *I*_*R*_
42	*α*-Terpinyl acetate	C_12_H_20_O_2_	1333	1333	1.35	—	—	MS, *I*_*R*_
43	Geranyl acetate	C_12_H_20_O_2_	1352	1352	—	—	3.75	MS, *I*_*R*_
44	*δ*-Elemene	C_15_H_24_	1365	1462	—	7.18	—	MS, *I*_*R*_
45	*β*-Elemene	C_15_H_24_	1398	1393	—	2.33	—	MS, *I*_*R*_
46	Bornyl isobutyrate	C_14_H_24_O_2_	1412	1402	—	1.03	—	MS, *I*_*R*_
47	*α*-Bergamotene	C_15_H_24_	1430	1430	—	—	0.43	MS, *I*_*R*_
48	*γ*-Muurolene	C_15_H_24_	1435	1435	—	—	0.63	MS, *I*_*R*_
49	*β*-*cis*-Farnesene	C_15_H_24_	1440	1446	—	—	0.92	MS, *I*_*R*_
50	(Z)-*β*-Farnesene	C_15_H_24_	1440	1443	—	**7.58**	—	MS, *I*_*R*_
51	*α*-Farnesene	C_15_H_24_	1458	1456	—	2.46	—	MS, *I*_*R*_
52	Alloaromadendrene	C_15_H_24_	1461	1461	0.97	—	—	MS, *I*_*R*_
53	Caryophyllene	C_15_H_24_	1494	1493	—	3.59	2.21	MS, *I*_*R*_
54	*α*-Muurolene	C_15_H_24_	1499	1500	—	2.97	—	MS, *I*_*R*_
55	*β*-Bisabolene	C_15_H_24_	1500	1500	—	—	0.59	MS, *I*_*R*_
56	Lavandulyl	C_15_H_26_O_2_	1504	1502	—	—	0.84	MS, *I*_*R*_
57	*γ*-Cadinene	C_15_H_24_	1513	1513	—	—	0.28	MS, *I*_*R*_
58	*δ*-Cadinene	C_15_H_24_	1524	1524	—	1.99	—	MS, *I*_*R*_
59	Trans-Nerolidol	C_15_H_26_O	1564	1563	—	2.81	—	MS, *I*_*R*_
60	Spathulenol	C_15_H_24_O	1575	1572	3.14	1.40	—	MS, *I*_*R*_
61	Caryophylene oxide	C_15_H_24_O	1581	1583	—	0.90	0.90	MS, *I*_*R*_
62	Germacrene D	C_15_H_24_	1588	1587	—	**9.17**	—	MS, *I*_*R*_
63	Carotol	C_15_H_26_O	1593	1594	—	1.60	—	MS, *I*_*R*_
64	Cedrenol	C_15_H_24_O	1604	1604	—	0.91	—	MS, *I*_*R*_
65	Caryophyllene oxide	C_15_H_24_O	1632	1630	—	1.63	—	MS, *I*_*R*_
66	*τ*-Muurolol	C_15_H_26_O	1641	1640	—	1.04	—	MS, *I*_*R*_
67	*τ*-Cadinol	C_15_H_26_O	1644	1640	—	—	0.59	MS, *I*_*R*_
68	*α*-Bisabolol	C_15_H_26_O	1682	1683	—	—	1.28	MS, *I*_*R*_
69	cis-Lanceol	C_15_H_24_O	1743	1746	—	0.84	—	MS, *I*_*R*_
70	Butanoic acid	C_15_H_22_O_2_	1773	1773	—	1.48	—	MS, *I*_*R*_

Total identified (%)					**99.98**	**95.82**	**97.47**	
Monoterpene hydrocarbons					57.18	17.24	8.61	
Oxygenated monoterpenes					38.73	27.67	62.49	
Sesquiterpene hydrocarbons					0.97	36.27	5.65	
Oxygenated sesquiterpenes					3.14	11.13	0.9	
Others					—	1.03	22.28	
Yield (%, v/w)					2.13	0.7	1.48	

^a^In order of elution on HP-5MS, ^b^components identified by RI and MS. ^c^Retention index determined from alkanes series (C9–C31). ^d^Retention index from data libraries (NIST) [30, 31]. Bold values represent the proportions of the major components for each oil.

**Table 2:** The MIC and MBC values of EPEO, LBEO, and OMEO against bacteria strains using microdilution assay.

Bacteria^a^	EPEO (%v/v)			LBEO (%v/v)			OMEO (%v/v)			Gentamicin^b^ (*μ*g/mL)		Kanamycin^b^ (*μ*g/mL)	
	MIC	MBC	Effect	MIC	MBC	Effect	MIC	MBC	Effect	MIC	MBC	MIC	MBC
*E. coli*	2	4	Bacteriostatic	1	1	Bactericidal	6	6	Bactericidal	16	32	32	32
*S. aureus*	0.25	0.25	Bactericidal	0.25	0.25	Bactericidal	0.5	0.5	Bactericidal	8	32	16	32

^a^Final bacterial concentration was 10^6^ CFU/mL. ^b^Gentamicin and kanamycin were used as references.

**Table 3:** Various combinations generated by the chosen mixture design and response data for each trial.

Number of experiments^a^	EPEO	OMEO	LBEO	MIC % (v/v)^b^	
				*E. coli*	*S. aureus*
1	1	0	0	2	0.25
2	0	1	0	6	0.5
3	0	0	1	1	0.25
4	0.5	0.5	0	2	0.25
5	0.5	0	0.5	2	0.25
6	0	0.5	0.5	2	0.25
7	0.333	0.333	0.333	0.5	0.125
8	0.333	0.333	0.333	0.5	0.125
9	0.333	0.333	0.333	0.5	0.125
10	0.667	0.167	0.167	1	0.125
11	0.167	0.667	0.167	2	0.25
12	0.167	0.167	0.667	0.5	0.125

^a^Experiments were carried out after randomization. ^b^Each response is the average of three replicates.

**Table 4:** Variance analysis for the three fitted models.

Models	*E. coli*					*S. aureus*				
	DF	SS	MS	*F*	*p* value	DF	SS	MS	*F*	*p* value
*R*	6	25.486212	4.24770	117.6945	<0.0001^*∗*^	6	0.125	0.021	29.41	0.001^*∗*^
*r*	5	0.180455	0.03609			5	0.004	0.001		
LOF	3	0.190	0.06015	14.32	0.07	3	0.0036	0.00122	12.2	0.051
PE	2	0.00012	0.0042			2	0.0001	0.0001		
Total	11	25.666667				11	0.129			

*R* ^2^	99%					97%				

*R*_adj_^2^	98%					93%				

*R*_pred_^2^	85%					42%				

*R*: regression; *r*: residual; LOF: lack of fit; PE: pure error; *R*^2^: coefficient of determination; adj: adjusted; pred: predicted; DF: degrees of freedom; SS: sum of squares; MS: mean square; ^*∗*^statistically significant.

**Table 5:** Estimated regression coefficients for the uncompleted cube regression model.

		*E. coli*			*S. aureus*		
Term	Coefficient	Estimation	*t*-ratio	*p* value	Estimation	*t*-ratio	*p* value
EPEO	*δ*_1_	2.02	10.99	0.0001^*∗*^	0.24	9.40	0.0002^*∗*^
OMEO	*δ*_2_	5.92	32.29	<0.0001^*∗*^	0.50	19.56	<0.0001^*∗*^
LBEO	*δ*_3_	0.97	5.29	0.0032^*∗*^	0.24	9.40	0.0002^*∗*^
EPEO^*∗*^ OMEO	*δ*_12_	−8.12	−8.79	0.0003^*∗*^	−0.51	−3.93	0.0111^*∗*^
EPEO ^*∗*^ LBEO	*δ*_13_	1.97	2.13	0.086	−0.03	−0.25	0.8158
OMEO ^*∗*^ LBEO	*δ*_23_	−6.21	−6.72	0.0011^*∗*^	−0.51	−3.93	0.0111^*∗*^
EPEO ^*∗*^ OMEO ^*∗*^ LBEO	*δ*_123_	−32.04	−6.38	0.0014^*∗*^	−2.70	−3.83	0.0123^*∗*^

^*∗*^Statistically significant at *p* < 0.05.

**Table 6:** FIC indices of the combined antibacterials against the tested bacterial strains.

Bacteria	Samples		MIC % (v/v)		FIC % (v/v)	FIC index	Effect
			Alone	Combination			
*S. aureus* ATCC 29213	1/2	EPEO	0.25	0.125	0.5	0.656	Partial synergy
	1/4	OMEO	0.5	0.0078	0.0156		
	1/4	EPEO	0.25	0.0625	0.25	0.281	Synergy
	1/32	OMEO	0.5	0.0156	0.0312		
	1/8	EPEO	0.25	0.3125	0.125	0.1874	Synergy
	1/16	OMEO	0.5	0.0312	0.0624		
	1/32	EPEO	0.25	0.007813	0.03125	0.156	Synergy
	1/8	OMEO	0.5	0.0625	0.125		
	1/64	EPEO	0.25	0.0039	0.0156	0.2656	Synergy
	1/4	OMEO	0.5	0.125	0.25		
	1/256	EPEO	0.25	0.00976	0.003907	0.5039	Partial synergy
	1/2	OMEO	0.5	0.25	0.25		
	1/2048	EPEO	0.25	0.0001220	0.00048828	0.25	Synergy
	1/4	LBEO	0.25	0.0625	0.25		
	1/4096	EPEO	0.25	6.103.10^−5^	0.00024412	0.5002	Partial synergy
	1/2	LBEO	0.25	0.125	0.5		
	1/4	LBEO	0.25	0.0625	0.25	0.75	Partial synergy
	1/2	OMEO	0.5	0.25	0.5		

*E. coli* ATCC 25922	1/2	EPEO	2	1	0.5	0.625	Partial synergy
	1/8	OMEO	6	0.75	0.125		
	1/4	EPEO	2	0.5	0.25	0.5	Synergy
	1/4	OMEO	6	1.5	0.25		
	1/4096	EPEO	2	0.00048828	0.00024414	0.50024414	Partial synergy
	1/2	OMEO	6	3	0.5		
	1/8	EPEO	2	0.25	0.125	0.625	Partial synergy
	1/2	LBEO	1	0.5	0.5		
	1/4	EPEO	2	0.5	0.25	0.25195313	Synergy
	1/512	L.BEO	1	0.00195313	0.00195313		
	1/2	EPEO	2	1	0.5	0.50024414	Partial synergy
	1/4096	LBEO	1	0.00024414	0.00024414		
	1/2	LBEO	1	0.5	0.5	0.625	Partial synergy
	1/8	OMEO	6	0.75	0.125		
	1/2	LBEO	1	0.25	0.25	0.5	Synergy
	1/4	OMEO	6	1.5	0.25		
	1/16	LBEO	1	0.0625	0.0625	0.5625	Partial synergy
	1/2	OMEO	6	3	0.5		

## Data Availability

All the data supporting the findings of this study are included in this article.

## Supplementary Materials

Supplementary Materials Supplement file 1: Chromatogram of gas chromatography (GC) analysis of *Ormenis mixta* EO. Supplement file 2: Chromatogram of gas chromatography (GC) analysis of *Eucalyptus polybractea* EO. Supplement file 3: Chromatogram of gas chromatography (GC) analysis of *Lavandula burnatii* EO.

## References

[1] Moukafih B., El Marrakchi S., Bennani I., Nchinech N., Achour S., El Kartouti A. (2022). New antibiotics for multi-drug resistant bacterial strains. *Cahiers Santé Médecine Thérapeutique*.

[2] Terreni M., Taccani M., Pregnolato M. (2021). New antibiotics for multidrug-resistant bacterial strains: latest research developments and future perspectives. *Molecules*.

[3] Angane M., Swift S., Huang K., Butts C. A., Quek S. Y. (2022). Essential oils and their major components: an updated review on antimicrobial activities, mechanism of action and their potential application in the food industry. *Foods*.

[4] Swamy M. K., Sinniah U. R. (2015). A comprehensive review on the phytochemical constituents and pharmacological activities of pogostemon cablin benth.: an aromatic medicinal plant of industrial importance. *Molecules*.

[5] Sharmeen J. B., Suroowan S., Rengasamy K R., Mahomoodally M F. (2020). chemistry, bioactivities, mode of action and industrial applications of essential oils. *Trends in Food Science & Technology*.

[6] Mahmud I., Shahria N., Yeasmin S. (2018). Ethnomedicinal, phytochemical and pharmacological profile of a mangrove plant Ceriops Decandra GriffDin Hou. *Journal of Complementary and Integrative Medicine*.

[7] Torres Neto L., Monteiro M. L. G., Machado M. A. M., Galvan D., Conte Junior C. A. (2022). An optimization of oregano, thyme, and lemongrass essential oil blend to simultaneous inactivation of relevant foodborne pathogens by simplex–centroid mixture design. *Antibiotics*.

[8] Chraibi M., Fadil M., Farah A., Lebrazi S., Fikri-Benbrahim K. (2021). Antimicrobial combined action of Mentha pulegium, Ormenis mixta and Mentha piperita essential oils against S. Aureus, E. Coli and C. Tropicalis: application of mixture design methodology. *Lebensmittel-Wissenschaft und-Technologie*.

[9] Mikolo B., Moyen R., Baloki N. T., Nguimbi E. (2020). Optimization by mixture design of the antimicrobial activities of five selected essential oils. *Journal of Medicinal Plants Research*.

[10] Chraibi M., Fadil M., Farah A., Benkhaira N., Lebrazi S., Fikri-Benbrahim K. (2023). Simplex-centroid design as innovative approach in the optimization of antimicrobial effect of Thymus satureioides, Myrtus communis and artemisia herba alba essential oils against Escherichia coli, Staphylococcus aureus and Candida tropicalis. *Experimental Parasitology*.

[11] Benkhaira N., Koraichi S. I., Fikri-Benbrahim K. (2021). Ethnobotanical survey on plants used by traditional healers to fight against COVID-19 in Fez city, northern Morocco. *Ethnobotany Research and Applications*.

[12] Benkhaira N., Ech-chibani N., Fikri-Benbrahim K. (2021). Ethnobotanical survey on the medicinal usage of two common medicinal plants in taounate region: artemisia herba-alba asso and Ormenis mixta (L.) dumort. *Ethnobotany Research and Applications*.

[13] El Hachlafi N., Benkhaira N., Ferioun M. (2022). Moroccan medicinal plants used to treat cancer: ethnomedicinal study and insights into pharmacological evidence. *Evidence-based Complementary and Alternative Medicine*.

[14] Bouyahya A., El Hachlafi N., Aanniz T. (2022). Natural bioactive compounds targeting histone deacetylases in human cancers: recent updates. *Molecules*.

[15] Shao J., Yin Z., Wang Y. (2020). Effects of different doses of Eucalyptus oil from Eucalyptus globulus labill on respiratory tract immunity and immune function in healthy rats. *Frontiers in Pharmacology*.

[16] Kokina M., Kalušević A., Nedović V. (2019). Characterization, antioxidant and antibacterial activity of essential oils and their encapsulation into biodegradable material followed by freeze drying. *Food Technology and Biotechnology*.

[17] King D. J., Gleadow R. M., Woodrow I. E. (2006). Regulation of oil accumulation in single glands of Eucalyptus polybractea. *New Phytologist*.

[18] Salehi B., Sharifi-Rad J., Quispe C. (2019). Insights into Eucalyptus genus chemical constituents, biological activities and health-promoting effects. *Trends in Food Science & Technology*.

[19] Sharma A. D., Kaur I. (2020). Jensenone from Eucalyptus essential oil as a potential inhibitor of COVID 19 corona virus infection. *Research & Reviews in Biotechnology & Biosciences*.

[20] Ouedrhiri W., Balouiri M., Bouhdid S., Harki E. H., Moja S., Greche H. (2018). Antioxidant and antibacterial activities of Pelargonium asperum and Ormenis mixta essential oils and their synergistic antibacterial effect. *Environmental Science and Pollution Research*.

[21] Yamada K., Mimaki Y., Sashida Y. (2005). Effects of inhaling the vapor of Lavandula burnatii super-derived essential oil and linalool on plasma adrenocorticotropic hormone (ACTH), catecholamine and gonadotropin levels in experimental menopausal female rats. *Biological & Pharmaceutical Bulletin*.

[22] Aly M. M., Al-Ghamdi M., Bafeel S. O., Khedr A. M. (2013). Antimicrobial qctivities and phytochemical analysis of the essential oil of Lavandula dentata and plectranthus tenuiflorus, collected from Al baha region, Saudi arabia. *Life Science Journal*.

[23] Simic N., Palic R., Vajs V., Milosavljevic S., Djokovic D. (2002). Composition and antibacterial activity of Achillea asplenifolia essential oil. *Journal of Essential Oil Research*.

[24] Hsouna A. B., Halima N. B., Abdelkafi S., Hamdi N. (2013). Essential oil from artemisia phaeolepis: chemical composition and antimicrobial activities. *Journal of Oleo Science*.

[25] Wang W., Li N., Luo M., Zu Y., Efferth T. (2012). Antibacterial activity and anticancer activity of Rosmarinus officinalis L. Essential oil compared to that of its main components. *Molecules*.

[26] Jianu C., Pop G., Gruia A. T., Horhat F. G. (2013). Chemical composition and antimicrobial activity of essential oils of lavender (Lavandula angustifolia) and lavandin (Lavandula x intermedia) grown in western Romania. *International Journal of Agriculture and Biology*.

[27] Melito S., Petretto G. L., Podani J. (2016). Altitude and climate influence Helichrysum italicum subsp. microphyllum essential oils composition. *Industrial Crops and Products*.

[28] Talbaoui A., Jamaly N., Aneb M. (2012). Chemical composition and antibacterial activity of essential oils from six Moroccan plants. *Journal of Medicinal Plants Research*.

[29] Zrira S., Elamrani A., Benjilali B. (2003). Chemical composition of the essential oil of pistacia lentiscus L. From Morocco—a seasonal variation. *Flavour and Fragrance Journal*.

[30] Babushok V. I., Linstrom P. J., Zenkevich I. G. (2011). Retention indices for frequently reported compounds of plant essential oils. *Journal of Physical and Chemical Reference Data*.

[31] Adams R. P. (2017). *Identification of Essential Oil Components by Gas Chromatography/Mass Spectrometry*.

[32] Benkhaira N., Koraichi S. I., Fikri-Benbrahim K. (2022). Vitro methods to study antioxidant and some biological activities of essential oils: a review. *Biointerface Res. Appl. Chem.*.

[33] Jeddi M., Fikri-Benbrahim K., El Hachlafi N., Benkhaira N., Aboussemdai A., Ouaritini Z. B. (2023). Chemical composition of thymus vulgaris, origanum compactum and vetiveria zizanoides essential oils and their antibacterial and antioxidant activities. *Tropical Journal of Natural Product Research*.

[34] El Hachlafi N., Benkhaira N., Al-Mijalli S. H. (2023). Phytochemical analysis and evaluation of antimicrobial, antioxidant, and antidiabetic activities of essential oils from Moroccan medicinal plants: Mentha suaveolens, Lavandula stoechas, and ammi visnaga. *Biomedicine & Pharmacotherapy*.

[35] Basri D. F., Luoi C. K., Azmi A. M., Latip J. (2012). Evaluation of the combined effects of stilbenoid from shorea gibbosa and vancomycin against methicillin-resistant Staphylococcus aureus (MRSA). *Pharmaceuticals*.

[36] Jeyaseeli L., Dasgupta A., Dastidar S. G., Molnar J., Amaral L. (2012). Evidence of significant synergism between antibiotics and the antipsychotic, antimicrobial drug flupenthixol. *European Journal of Clinical Microbiology & Infectious Diseases*.

[37] Goupy J., Creighton L. (2006). *Introduction Aux Plans D’expériences-3ème Édition-Livre+ CD-Rom*.

[38] Kachkoul R., Benjelloun Touimi G., Bennani B. (2021). The synergistic effect of three essential oils against bacteria responsible for the development of lithiasis infection: an optimization by the mixture design. *Evidence-based Complementary and Alternative Medicine*.

[39] Mahmud J., Muranyi P., Salmieri S., Lacroix M. (2023). Optimization of a natural antimicrobial formulation against potential meat spoilage bacteria and food-borne pathogens: mixture design methodology and predictive modeling. *Microbial Pathogenesis*.

[40] Aouan B., El Alouani M., Alehyen S. (2022). Application of central composite design for optimisation of the development of metakaolin based geopolymer as adsorbent for water treatment. *International Journal of Environmental Analytical Chemistry*.

[41] Benkhaira N., Zouine N., Fadil M. (2023). Application of mixture design for the optimum antibacterial action of chemically-analyzed essential oils and investigation of the antiadhesion ability of their optimal mixtures on 3D printing material. *Bioprinting*.

[42] Wan Hassan W. N. F., Ismail M. A., Lee H.-S. (2020). Mixture optimization of high-strength blended concrete using central composite design. *Construction and Building Materials*.

[43] Fadil M., Fikri-Benbrahim K., Rachiq S. (2018). Combined treatment of Thymus vulgaris L., Rosmarinus officinalis L. And Myrtus communis L. Essential oils against Salmonella typhimurium: optimization of antibacterial activity by mixture design methodology. *European Journal of Pharmaceutics and Biopharmaceutics*.

[44] Kursuncu B., Gencel O., Bayraktar O. Y., Shi J., Nematzadeh M., Kaplan G. (2022). Optimization of foam concrete characteristics using response surface methodology and artificial neural networks. *Construction and Building Materials*.

[45] Soussi M., Fadil M., Yaagoubi W. A., Benjelloun M., El Ghadraoui L. (2022). Simultaneous optimization of phenolic compounds and antioxidant abilities of Moroccan pimpinella anisum extracts using mixture design methodology. *Processes*.

[46] Vera Candioti L., De Zan M. M., Cámara M. S., Goicoechea H. C. (2014). Experimental design and multiple response optimization. Using the desirability function in analytical methods development. *Talanta*.

[47] Ambrož M., Matoušková P., Skarka A., Zajdlová M., Žáková K., Skálová L. (2017). The effects of selected sesquiterpenes from myrica rubra essential oil on the efficacy of doxorubicin in sensitive and resistant cancer cell lines. *Molecules*.

[48] Sadgrove N. J., Padilla-González G. F., Phumthum M. (2022). Fundamental chemistry of essential oils and volatile organic compounds, methods of analysis and authentication. *Plants*.

[49] Finefield J. M., Sherman D. H., Kreitman M., Williams R. M. (2012). Enantiomeric natural products: occurrence and biogenesis. *Angewandte Chemie International Edition*.

[50] Fahad A., Flematti G., Hammer K. (2018). Antimicrobial activity of several cineole-rich western Australian Eucalyptus essential oils. *Microorganisms*.

[51] Juan L. W., Lucia A., Zerba E. N., Harrand L., Marco M., Masuh H. M. (2011). Chemical composition and fumigant toxicity of the essential oils from 16 species of Eucalyptus against haematobia irritans (Diptera: muscidae) adults. *Journal of Economic Entomology*.

[52] Akhtar M., Ahmad R., Qureshi T. M., Iqbal Z., Akhter J. (2011). Variation in composition and yield of foliage oil of Eucalyptus polybractea. *Journal of the Chemical Society of Pakistan*.

[53] Goodger J. Q., Woodrow I. E. (2008). Selection gains for essential oil traits using micropropagation of Eucalyptus polybractea. *Forest Ecology and Management*.

[54] Gilles M., Zhao J., An M., Agboola S. (2010). Chemical composition and antimicrobial properties of essential oils of three Australian Eucalyptus species. *Food Chemistry*.

[55] Hajdari A., Mustafa B., Nebija D., Miftari E., Quave C. L., Novak J. (2015). Chemical composition of juniperus communis L. Cone essential oil and its variability among wild populations in kosovo. *Chemistry and Biodiversity*.

[56] Leontaritou P., Lamari F. N., Papasotiropoulos V., Iatrou G. (2020). Morphological, genetic and essential oil variation of Greek sage (salvia fruticosa mill.) populations from Greece. *Industrial Crops and Products*.

[57] Elouaddari A., El Amrani A., Eddine J. J. (2013). Yield and chemical composition of the essential oil of Moroccan chamomile [cladanthus mixtus (L.) chevall.] growing wild at different sites in Morocco. *Flavour and Fragrance Journal*.

[58] Satrani B., Ghanmi M., Farah A., Aafi A., Fougrach H., Bourkhiss B. (2007). Composition Chimique et Activité Antimicrobienne de l’huile Essentielle de Cladanthus Mixtus. *Bulletin de la Société de Pharmacie de Bordeaux*.

[59] Lesage-Meessen L., Bou M., Sigoillot J.-C., Faulds C. B., Lomascolo A. (2015). Essential oils and distilled straws of lavender and lavandin: a review of current use and potential application in white Biotechnology. *Applied Microbiology and Biotechnology*.

[60] Bajalan I., Rouzbahani R., Pirbalouti A. G., Maggi F. (2017). Chemical composition and antibacterial activity of Iranian Lavandula× hybrida. *Chemistry and Biodiversity*.

[61] Caputo L., Souza L. F., Alloisio S., Cornara L., De Feo V. (2016). Coriandrum sativum and Lavandula angustifolia essential oils: chemical composition and activity on central nervous system. *International Journal of Molecular Sciences*.

[62] Garzoli S., Turchetti G., Giacomello P., Tiezzi A., Laghezza Masci V., Ovidi E. (2019). Liquid and vapour phase of lavandin (Lavandula× intermedia) essential oil: chemical composition and antimicrobial activity. *Molecules*.

[63] Al-Mijalli S. H., Mrabti H. N., El Hachlafi N. (2023). Integrated analysis of antimicrobial, antioxidant, and phytochemical properties of cinnamomum verum: a comprehensive in vitro and in silico study. *Biochemical Systematics and Ecology*.

[64] Singh S., Tewari G., Pande C., Singh C. (2013). Variation in essential oil composition of Ocimum americanum L. From north-western himalayan region. *Journal of Essential Oil Research*.

[65] Hajdari A., Mustafa B., Kaçiku A. (2016). Chemical composition of the essential oil, total phenolics, total flavonoids and antioxidant activity of methanolic extracts of satureja Montana L. *Records of Natural Products*.

[66] El Omari N., Charfi S., Elmenyiy N. (2022). Essential oils for combating antimicrobial resistance: mechanism insights and clinical uses. *Antimicrobial Resistance: Underlying Mechanisms and Therapeutic Approaches*.

[67] Hugo W. B., Longworth A. R. (2011). Some aspects of the mode of action of chlorhexidine. *Journal of Pharmacy and Pharmacology*.

[68] Burt S. (2004). Essential oils: their antibacterial properties and potential applications in foods—a review. *International Journal of Food Microbiology*.

[69] Bouyahya A., Et-Touys A., Bakri Y. (2017). Chemical composition of Mentha pulegium and Rosmarinus officinalis essential oils and their antileishmanial, antibacterial and antioxidant activities. *Microbial Pathogenesis*.

[70] Hashemi S. M. B., Jafarpour D. (2020). Synergistic properties of Eucalyptus caesia and Dracocephalum multicaule montbr & auch essential oils: antimicrobial activity against food borne pathogens and antioxidant activity in pear slices. *Journal of Food Processing and Preservation*.

[71] Bassolé I. H. N., Juliani H. R. (2012). Essential oils in combination and their antimicrobial properties. *Molecules*.

[72] Bouyahya A., Et-Touys A., Abrini J. (2017). Lavandula stoechas essential oil from Morocco as novel source of antileishmanial, antibacterial and antioxidant activities. *Biocatalysis and Agricultural Biotechnology*.

[73] Dadalioğlu I., Evrendilek G. A. (2004). Chemical compositions and antibacterial effects of essential oils of Turkish oregano (origanum minutiflorum), bay laurel (laurus nobilis), Spanish lavender (Lavandula stoechas L.), and fennel (foeniculum vulgare) on common foodborne pathogens. *Journal of Agricultural and Food Chemistry*.

[74] Cherrat L., Espina L., Bakkali M., Pagán R., Laglaoui A. (2014). Chemical composition, antioxidant and antimicrobial properties of Mentha pulegium, Lavandula stoechas and satureja calamintha scheele essential oils and an evaluation of their bactericidal effect in combined processes. *Innovative Food Science & Emerging Technologies*.

[75] Silva V. A., Sousa J. P., Guerra F. Q. S. (2015). Antibacterial activity of Ocimum basilicum essential oil and linalool on bacterial isolates of clinical importance. *International Journal of Pharmacognosy and Phytochemical Research*.

[76] Hussain A. I., Anwar F., Hussain Sherazi S. T., Przybylski R. (2008). Chemical composition, antioxidant and antimicrobial activities of basil (Ocimum basilicum) essential oils depends on seasonal variations. *Food Chemistry*.

[77] Bouchra C., Achouri M., Idrissi Hassani L., Hmamouchi M. (2003). Chemical composition and antifungal activity of essential oils of seven Moroccan labiatae against botrytis cinerea pers: Fr. *Journal of Ethnopharmacology*.

[78] Djenane D., Yangüela J., Amrouche T., Boubrit S., Boussad N., Roncalés P. (2011). Chemical composition and antimicrobial effects of essential oils of Eucalyptus globulus, Myrtus communis and satureja hortensis against Escherichia coli O157: H7 and Staphylococcus aureus in minced beef. *Food Science and Technology International*.

[79] Oyedeji A., Ekundayo O., Olawore O. N., Adeniyi B. A., Koenig W. A. (1999). Antimicrobial activity of the essential oils of five Eucalyptus species growing in Nigeria. *Fitoterapia*.

[80] Assaggaf H. M., Naceiri Mrabti H., Rajab B. S. (2022). Singular and combined effects of essential oil and honey of Eucalyptus globulus on anti-inflammatory, antioxidant, dermatoprotective, and antimicrobial properties: in vitro and in vivo findings. *Molecules*.

[81] Bouhdid S., Abrini J., Zhiri A., Espuny M. J., Manresa A. (2009). Investigation of functional and morphological changes in Pseudomonas aeruginosa and Staphylococcus aureus cells induced by origanum compactum essential oil. *Journal of Applied Microbiology*.

[82] Bouhdid S., Abrini J., Amensour M., Zhiri A., Espuny M. J., Manresa A. (2010). Functional and ultrastructural changes in Pseudomonas aeruginosa and Staphylococcus aureus cells induced by cinnamomum verum essential oil. *Journal of Applied Microbiology*.

[83] Bouyahya A., Bakri Y., Et-Touys A. (2017). Resistance to antibiotics and mechanisms of action of essential oils against bacteria. *Phytothérapie*.

[84] Myszka K., Schmidt M. T., Majcher M., Juzwa W., Olkowicz M., Czaczyk K. (2016). Inhibition of quorum sensing-related biofilm of Pseudomonas fluorescens KM121 by Thymus vulgare essential oil and its major bioactive compounds. *International Biodeterioration & Biodegradation*.

[85] Luís Â., Duarte A., Gominho J., Domingues F., Duarte A. P. (2016). Chemical composition, antioxidant, antibacterial and anti-quorum sensing activities of Eucalyptus globulus and Eucalyptus radiata essential oils. *Industrial Crops and Products*.

[86] Zieniuk B., Bętkowska A. Mixture design as a tool for optimization of antimicrobial activity of selected essential oils.

[87] Souiy Z., Elaissi A., Jlassi I. (2021). Application of simplex-centroid design methodologies to optimize the anti-bacterial and anti-candidal activity of the mixture of menthapulegium, pituranthos chloranthus and Thymus algeriensis essential oils. *Medicinal & Aromatic Plants*.

[88] Hyldgaard M., Mygind T., Meyer R. L. (2012). Essential oils in food preservation: mode of action, synergies, and interactions with food matrix components. *Frontiers in Microbiology*.

[89] Pei R., Zhou F., Ji B., Xu J. (2009). Evaluation of combined antibacterial effects of eugenol, cinnamaldehyde, thymol, and carvacrol against E. Coli with an improved method. *Journal of Food Science*.

[90] Silva J. D. A., Nascimento M. G. P., Grazina L. G., Castro K. N. C., Mayo S. J., Andrade I. M. (2015). Ethnobotanical survey of medicinal plants used by the community of sobradinho, lus correia, piau, Brazil. *Journal of Medicinal Plants Research*.

[91] Ultee A., Slump R. A., Steging G., Smid E. J. (2000). Antimicrobial activity of carvacrol toward Bacillus cereus on rice. *Journal of Food Protection*.

[92] Bassolé I. H. N., Lamien-Meda A., Bayala B. (2010). Composition and antimicrobial activities of lippia multiflora moldenke, Mentha x piperita L. And Ocimum basilicum L. Essential oils and their major monoterpene alcohols alone and in combination. *Molecules*.

[93] Tserennadmid R., Takó M., Galgóczy L. (2011). Anti yeast activities of some essential oils in growth medium, fruit juices and milk. *International Journal of Food Microbiology*.

[94] Vuuren S. F. V., Viljoen A. M. (2007). Antimicrobial activity of limonene enantiomers and 1, 8‐cineole alone and in combination. *Flavour and Fragrance Journal*.

[95] García‐García R., López‐Malo A., Palou E. (2011). Bactericidal action of binary and ternary mixtures of carvacrol, thymol, and eugenol against Listeria innocua. *Journal of Food Science*.

[96] Pina‐Vaz C., Gonçalves Rodrigues A., Pinto E. (2004). Antifungal activity of Thymus oils and their major compounds. *Journal of the European Academy of Dermatology and Venereology*.

[97] Mahmoudi R., Katiraee F., Tajik H., Abbas A., Farshid, Omid F. (2016). Inhibitory effect of Mentha longifolia L. Essential oil against Listeria monocytogenes using transmission electron microscopy. *International Journal of Veterinary Sciences Research*.

[98] Inouye S., Takizawa T., Yamaguchi H. (2001). Antibacterial activity of essential oils and their major constituents against respiratory tract pathogens by gaseous contact. *Journal of Antimicrobial Chemotherapy*.

[99] Ultee A., Bennik M. H. J., Moezelaar R. (2002). The phenolic hydroxyl group of carvacrol is essential for action against the food-borne pathogen *Bacillus cereus*. *Applied and Environmental Microbiology*.

[100] Vieira M., Bessa L. J., Martins M. R. (2017). Chemical composition, antibacterial, antibiofilm and synergistic properties of essential oils from Eucalyptus globulus labill. And seven mediterranean aromatic plants. *Chemistry and Biodiversity*.

[101] Moussii I. M., Nayme K., Timinouni M., Jamaleddine J., Filali H., Hakkou F. (2020). Synergistic antibacterial effects of Moroccan artemisia herba alba, Lavandula angustifolia and Rosmarinus officinalis essential oils. *Synergy*.

[102] Santiesteban‐López A., Palou E., López‐Malo A. (2007). Susceptibility of food‐borne bacteria to binary combinations of antimicrobials at selected aw and PH. *Journal of Applied Microbiology*.

[103] Saeedi S., Sabbagh S. K., Bazi S. (2014). Antibacterial Effect of Eucalyptus Globules against Resistance Staphyloccocus Aureus.

[104] Mashwani Z. U. R., Khan T., Khan M. A., Nadhman A. (2015). Synthesis in plants and plant extracts of silver nanoparticles with potent antimicrobial properties: current status and future prospects. *Applied Microbiology and Biotechnology*.

[105] Yammine J., Chihib N.-E., Gharsallaoui A., Dumas E., Ismail A., Karam L. (2022). Essential oils and their active components applied as: free, encapsulated and in hurdle Technology to fight microbial contaminations. A review. *Heliyon*.

[106] Chouhan S., Sharma K., Guleria S. (2017). Antimicrobial activity of some essential oils—present status and future perspectives. *Medicine (Baltimore)*.

[107] Reichling J., Suschke U., Schneele J., Geiss H. K. (2006). Antibacterial activity and irritation potential of selected essential oil components–structure-activity relationship. *Natural Product Communications*.

