# Supplementary material for: Combination of Chemically-Characterized Essential Oils from Eucalyptus polybractea, Ormenis mixta, and Lavandula burnatii: Optimization of a New Complete Antibacterial Formulation Using Simplex-Centroid Mixture Design
Source: Adv Pharmacol Pharm Sci. 2023 Aug 21;2023:5593350. doi: 10.1155/2023/5593350 (PMC10462449; doi:10.1155/2023/5593350)
Supplement: Supplementary Materials — Supplement file 1: Chromatogram of gas chromatography (GC) analysis of Ormenis mixta EO. Supplement file 2: Chromatogram of gas chromatography (GC) analysis of Eucalyptus polybractea EO. Supplement file 3: Chromatogram of gas chromatography (GC) analysis of Lavandula burnatii EO. [file 5593350.f1.docx]

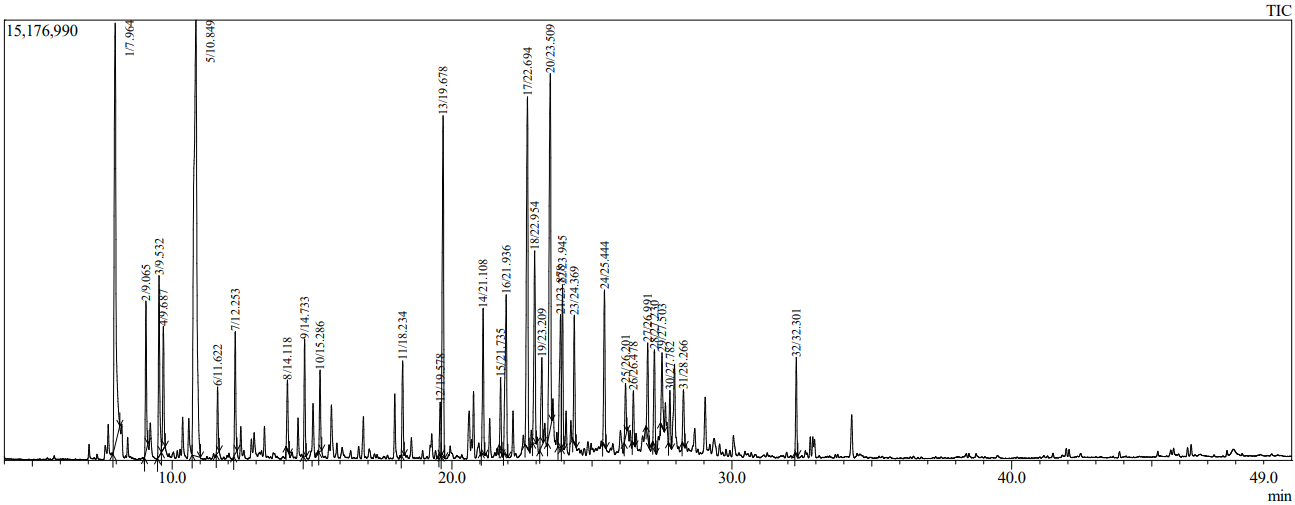


**Supplement file 1.** Chromatogram of gas chromatography (GC) analysis of *Ormenis mixta* EO*.*


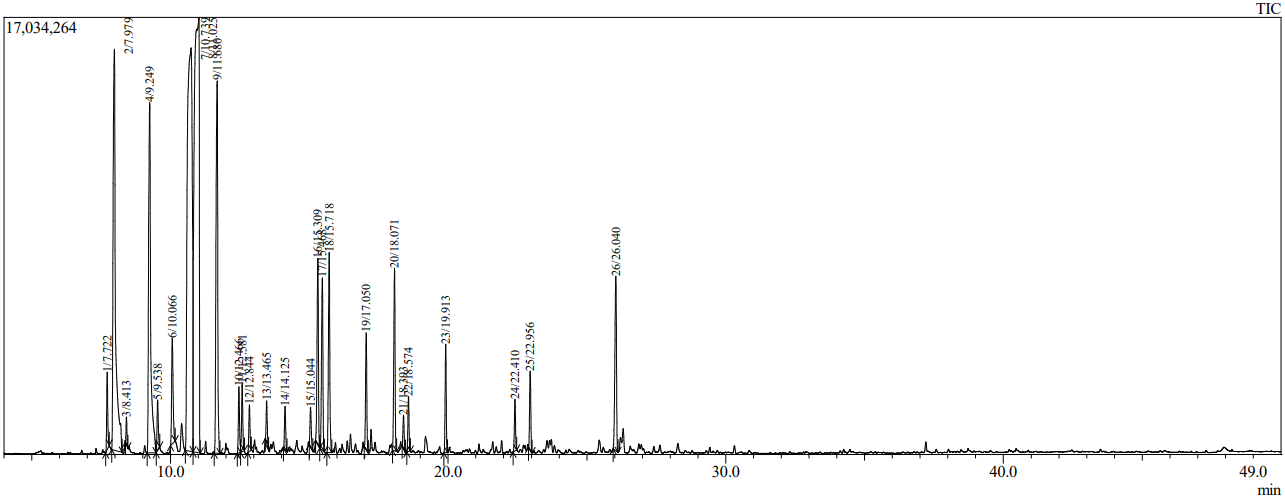


**Supplement file 2.** Chromatogram of gas chromatography (GC) analysis of *Eucalyptus polybractea* EO*.*


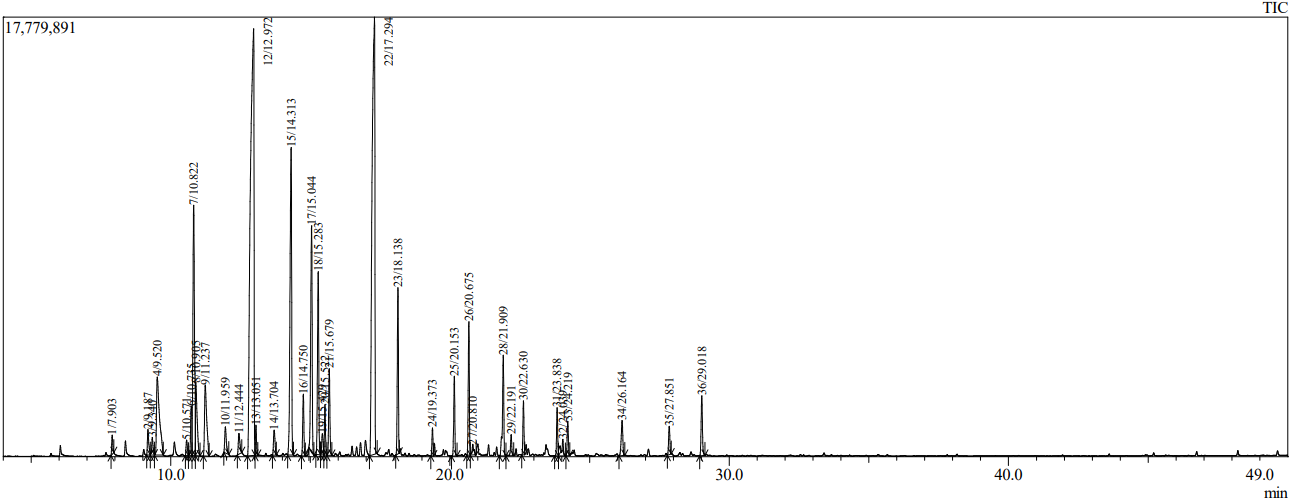


**Supplement file 3.** Chromatogram of gas chromatography (GC) analysis of *Lavandula burnatii* EO*.*
